# Deletion of metal transporter Zip14 reduces major histocompatibility complex II expression in murine small intestinal epithelial cells

**DOI:** 10.1073/pnas.2422321121

**Published:** 2024-12-30

**Authors:** Felix R. Jimenez-Rondan, Courtney H. Ruggiero, Alberto Riva, Fahong Yu, Lauren S. Stafford, Tyler R. Cross, Joseph Larkin, Robert J. Cousins

**Affiliations:** ^a^Center for Nutritional Sciences, Food Science and Human Nutrition Department, College of Agricultural and Life Sciences, University of Florida, Gainesville, FL 32611; ^b^Bioinformatics Core Facility, Interdisciplinary Center for Biotechnology Research, University of Florida, Gainesville, FL 32611; ^c^Department of Microbiology and Cell Science, College of Agricultural and Life Sciences, University of Florida, Gainesville, FL 32611; ^d^Department of Biochemistry and Molecular Biology, College of Medicine, University of Florida, Gainesville, FL 32611

**Keywords:** zinc, transport, intestinal immunity, epigenetic, Akkermansia

## Abstract

Documented worldwide, impaired immunity is a cardinal signature resulting from loss of dietary zinc, an essential micronutrient. A steady supply of zinc to meet cellular requirements is regulated by an array of zinc transporters. Deletion of the transporter Zip14 (Slc39a14) in mice produced intestinal inflammation. Elevated fecal lipocalin-2, calprotectin, IgG levels, and dysbiosis support the inflammatory phenotype. Here, we show through RNA-sequencing, using purified intestinal epithelial cells (IECs), that *Zip14* deletion produces markedly reduced expression of major histocompatibility complex class II (MHCII) molecules and the master MHCII transactivator (*Ciita*). qPCR, western analysis, and immunohistochemistry confirmed loss of MHCII. Spectrofluorimetry with zinc probe FluoZin-3 showed reduced labile zinc in IECs from knockout mice. Chromatin immunoprecipitation assays, using Ciita antibody and IEC chromatin, suggest decreased transcription accounts for depressed expression of specific MHCII genes. Assay for Transposase-Accessible Chromatin (ATAC) sequencing (ATAC-seq) demonstrated that *H2-Aa*, *H2-Ab1* and other MHCII genes result from chromatin remodeling yielding closed chromatin at regulatory regions of these genes. In agreement, ATAC-seq showed peak density of the chromosomal regulatory region of Ciita is consistent with down regulation of specific MHCII genes in IECs with *Zip14* loss. Finally, dietary zinc supplementation of knockout mice and zinc supplementation of intestinal organoids with *Zip14* deletion restored transcript levels. Taken together, our data suggest that cellular zinc delivery, via Zip14, is necessary for proper chromatin occupancy, required for normal MHCII expression and effective immune functions, and to preclude inflammatory disorders of the small intestine.

## Significance

Zinc deficiency can result in immune disorders of the intestinal tract in humans and domestic animals. Mechanisms responsible are unclear, as are those for responsiveness to zinc supplementation interventions. In vivo and in vitro experiments reported here, using purified intestinal epithelial cells and intestinal organoids, show that zinc withdrawal through genetic deletion of a zinc transporter produced an inflammatory phenotype and a reduction in the production of major histocompatibility complex proteins class II (MHCII) through transcriptional reprogramming of cellular chromatin. MHCII proteins are necessary for intestinal defense, such as food allergy and inflammatory disorders. We further showed that zinc supplementation in vivo and in vitro restored expression of specific MHCII genes to within normal levels.

Zinc is an essential micronutrient for all living organisms. In animals, a lack of dietary zinc is associated with growth stunting, infections, behavioral defects, and gastrointestinal disease, including endemic diarrhea and inflammation. These disorders have health implications worldwide (1, 2). Despite the well-defined structural, catalytic, and regulatory roles for zinc in biology, the underlying biochemical defects responsible for zinc-related disorders remain elusive (3).

For humans, intake of zinc from diets is highly variable. Fortunately, zinc metabolism is tightly controlled through homeostatic mechanisms. Key to the effectiveness of those mechanisms are the two protein families ZIP (SLC39A) and ZnT (SLC30A), composed of 23 proteins that contribute to cellular zinc transport (4, 5). Expression of these proteins is regulated by transcriptional and/or posttranslational processes. Some of the transporter genes respond to dietary zinc status and/or physiological stimuli. Our interest has focused on Zip14, a divalent metal transporter (Slc39a14) that is highly expressed in the small intestine and liver at steady state. Hepatic *Zip14* expression in mice is stimulated by proinflammatory mediators (6, 7), and ER stress (8). These events are accompanied by concurrent measurable changes in zinc transport, thus demonstrating that *Zip14* expression is physiologically regulated when cellular zinc needs must be augmented.

To evaluate a possible function for Zip14 in delivering zinc for cellular needs, we used a mouse strain with a whole-body *Zip14* ablation (*KO*). The phenotype for those mice includes increased intestinal permeability and inflammation (9). *Zip14* expression is very high in the proximal small intestine (9), where minimally digested food components are abundant. In order to focus more closely on the Zip14 phenotype in the small intestine, we developed a murine strain with an enterocyte-specific deletion of Zip14 (*Zip14*^*ΔIEC*^). Using intestinal epithelial cells (IECs) and intestinal organoids derived from that *Zip14*^*ΔIEC*^ strain, we showed altered transcription factor binding to regulatory regions of specific differentially expressed genes (DEGs) that could account for some of the demonstrated phenotype of *Zip14* deletion (10). We hypothesized that loss of Zip14-mediated zinc transport altered zinc-dependent HDAC (histone deacetylase) activity leading to differential expression of specific genes through an epigenetic mechanism.

Inflammatory gastrointestinal disorders are among the signatures of malfunctioning zinc metabolism in humans and domestic animals (11–22). Some of those have been reported as treatable with supplemental zinc. Here, we report RNA-sequencing (RNA-seq) and Assay for Transposase-Accessible Chromatin (ATAC) sequencing (ATAC-seq) experiments, using highly purified IECs, showed *Zip14* deletion led to a pronounced reduction in expression of major histocompatibility complex class II (MHCII) and Ciita, the master regulator required for MHCII expression (23). Our findings demonstrate that Zip14 is essential for transcriptional programming in intestinal epithelial cells needed for maximal immune defense processes, particularly those requiring MHCII expression. These results may explain how one mechanism of nutritional zinc deficiency contributes to defective intestinal immunity. The mechanism is zinc-responsive, therefore, selective zinc supplementation may aid in some intestinal disorders involving MHCII.

## Results

### Influence of Zip14 Deletion Results in Intestinal Inflammation and Dysbiosis.

**Fig. 1.**
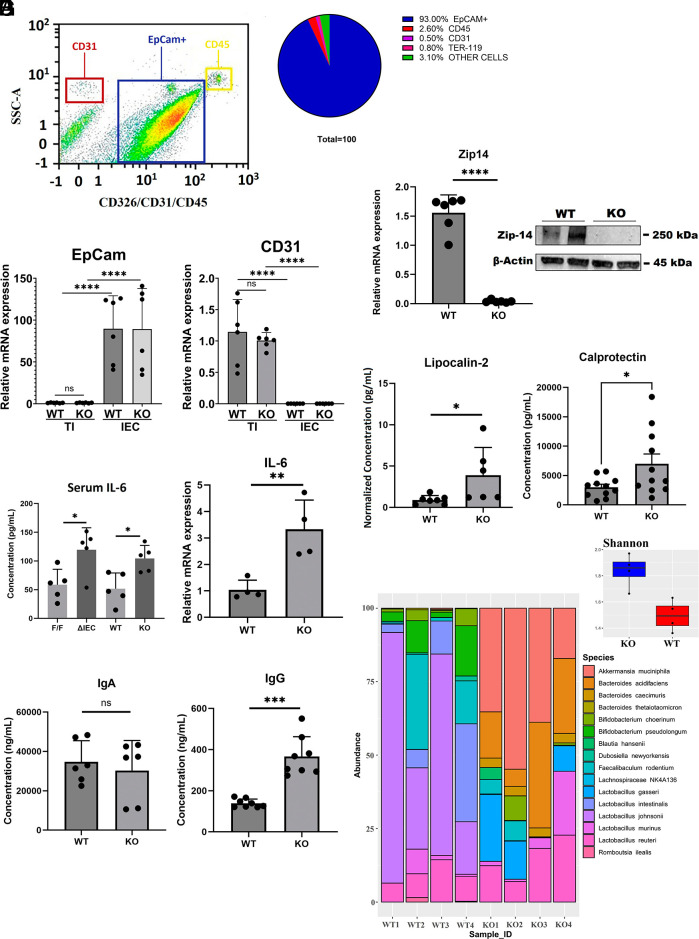
Inflammatory phenotype of enterocytes produced through *Zip14* ablation. (*A*) Representative flow plots of EpCAM+ with minimal CD31 and CD45 in purified Intestinal epithelial cells (IECs) from proximal small intestine obtained by flow cytometry (*Left*) with pie chart summary (*Right*). (*B*) Relative abundance of *EpCAM* and *CD31* mRNAs in total small intestine and purified IECs from wild type (*WT*) and knockout (*KO*) mice, measured by qPCR (n = 6 mice/group). (*C*) *Zip14* mRNA (qPCR assay, n = 6 mice/group) and ZIP14 protein (western analysis, n = 2 mice/group) in IECs from *WT* and *KO* mice demonstrating loss of ZIP14 in IECs. (*D*) Serum IL-6 concentrations of both *Zip14^F/F^* and *Zip14*^*ΔIEC*^ and *WT* and *KO* mice (n = 5 mice/group); IL-6 transcript levels of IECs from *WT* and *KO* mice (n = 4 mice/group). (*E*) Fecal lipocalin-2 and calprotectin concentrations of *WT* and *KO* mice (n = 6 to 11 mice/group). (*F*) IgA and IgG concentrations in feces of *WT* and *KO* mice (n = 6 to 8 mice/group). (*G*) Microbial 16S rRNA sequencing of feces from *WT* and *KO* mice showing species and alpha diversity (Shannon plot) (n = 4 mice/group). ∗P<0.05, ∗∗P<0.01, ∗∗∗P<0.001, and ∗∗∗∗P<0.0001.

Purity of the IEC preparations used was evaluated by FACS analysis. EpCAM+ cells represented 93% of the total cell population demonstrating high abundance of enterocytes (Fig. 1*A*). Only small amounts of CD45+ (2.6%), CD31+ (0.5%), TER-119+ (0.8%), and unmarked (3.1%) cells were detected. qPCR of RNA from the purified IECs substantiated the high abundance of enterocytes, based on FACS distribution (Fig. 1*B*). Specifically, there is greater abundance of EpCAM and lesser abundance of T cell marker, Cd31, in the IECs relative to expression of these transcripts in total intestine. Furthermore, relative Ct values from qPCR for EpCAM compared to 18S demonstrated there was a purification factor of >800% of the IEC preparation compared to total intestine. We also determined transcript abundances for other IEC-specific genes, i.e. *Lgr5*, *BML1*, *Alpi*, and *Akp3* relative to total intestine (*SI Appendix*, Fig. S1). Verification of the *Zip14* deletion in the IEC population was demonstrated with the lack of Zip14 mRNA and protein in tissue from the *KO* mice (Fig. 1*C*). In contrast, the *KO* mice had increased serum IL-6 concentrations as well as increased *IL-6* mRNA in IECs, suggestive of systemic and local inflammation (Fig. 1*D*). Inflammation markers, lipocalin-2 and calprotectin were increased in feces from *KO* mice, thus supporting intestinal inflammation as part of the Zip14 deficient phenotype (Fig. 1*E*). This conclusion is supported by the increased IgG levels without IgA changes in feces of the *KO* mice compared to the *WT* mice (Fig. 1*F* and *SI Appendix*, Fig. S2). *Zip14* ablation produced marked dysbiosis, based on 16S rRNA sequencing of fecal extracts (P<0.05). Specifically, the greater relative abundance of *Lactobacillus*_johnsonii species in *WT* mice feces was absent in feces from the *KO* mice (Fig. 1*G*). In contrast, Akkermansia_muniniphila was very abundant in feces from the *KO* mice, but was absent in feces from *WT* mice (Fig. 1*G*). A Shannon Plot shows the marked alpha diversity in fecal microbial species between the two genotypes (Fig. 1*G*). Commensal microbiota distribution and plots demonstrated wide differences between *WT* and *KO* samples (*SI Appendix*, Fig. S3 and Table S1).

### Loss of Zip14 Selectively Alters the Transcriptome of Enterocytes.

**Fig. 2.**
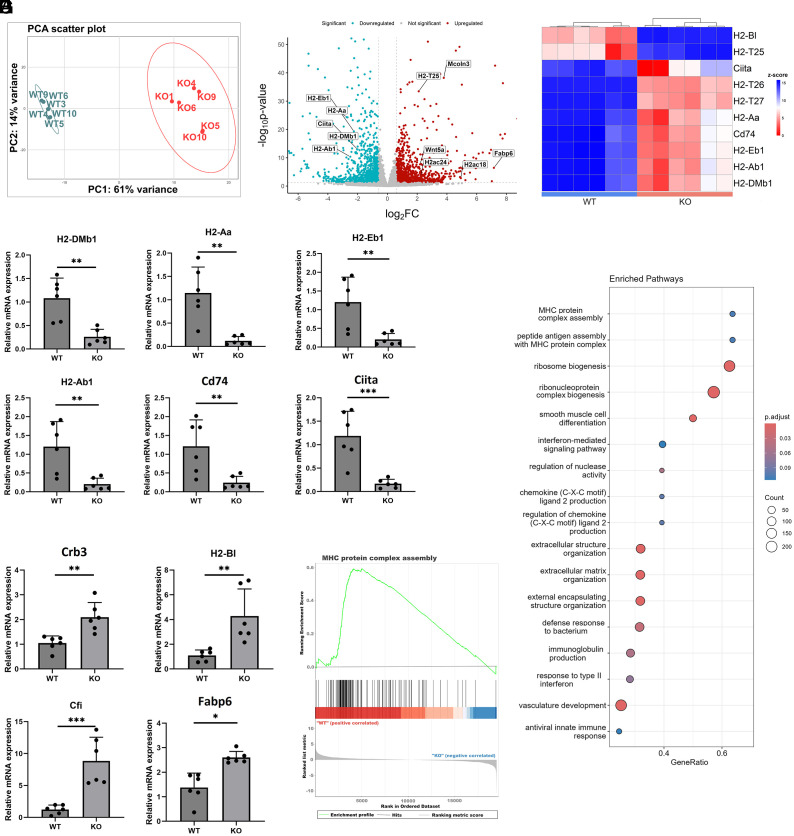
RNA-sequencing demonstrates reduced expression of major histocompatibility complex II (*MHCII*) mRNAs in purified IECs produced through *Zip14* ablation. (*A*) Principal-component analysis of transcript differences between *WT* and *KO* mice. Circles indicate P<0.05 for each genotype group (n = 6 mice/group). (*B*) Volcano plot of RNA-seq as fold-change (FC ± 2.0 to 8.0) showing up-regulated and down-regulated differentially expressed genes (DEGs). Specific genes are identified. (*C*) Heat map showing expression intensities of specific down-regulated MHCII-related transcripts of purified IECs from individual *WT* and *KO* mice. Each column represents transcript abundance from one individual mouse (n = 6 mice/group). (*D*) Individual qPCR assays confirming the relative transcript abundance in purified IECs, showing downregulation of *MHCII* genes and *Ciita*, using RNA from *WT* and *KO* mice (n = 6 mice/group). (*E*) Individual qPCR assays confirming the relative transcript abundance in purified IECs of some representative up-regulated genes using RNA from *WT* and *KO* mice (n = 6 mice/group). (*F*) Normalized gene set enrichment based on KEGG analysis (based on z-score) of RNA-seq dataset identifies MHC protein complex assembly among significantly down-regulated DEGs from purified IECs from *KO* mice. (*G*) Gene set enrichment analysis of MHC protein. ∗P<0.05, ∗∗P<0.01, and ∗∗∗P<0.001.

Our first goal was to examine the transcriptional changes related to *Zip14* ablation. Principal components analysis (PCA) of transcriptomic data derived from RNA-seq demonstrated a complete separation of the expression scores for DEGs between genotypes (Fig. 2*A*). This difference suggests that *Zip14* ablation was a primary factor producing the difference. RNA from IECs from both genotypes is summarized in the volcano plot of DEGs (Fig. 2*B*). Using a FC cutoff of ±2.0 (P<0.05), a total of 226 transcripts were up-regulated and 192 transcripts were down-regulated. The entire RNA-seq dataset is presented in *SI Appendix*, Table S2. We have focused on down-regulated genes reasoning that downregulation provides a reflection of defective zinc-dependent functions found in nutritional zinc deficiency. Surprisingly, RNA-seq revealed a robust array of MHCII genes that were markedly down-regulated as the result of the global *Zip14* deletion, as shown in the heatmap (Fig. 2*C*). These reductions in expression point to Zip14 as essential for maintenance of intestinal immune defense.

Differential expression of specific genes between the genotypes, identified by RNA-seq above, was confirmed with qPCR, using RNA from purified IECs. Highly relevant to inflammation were the confirmed markedly decreased expression of *H2-DMb1*, *H2-Aa*, *H2-Eb1*, *H2-Ab1*, *Cd74*, and *Ciita*, the MHCII regulator (Fig. 2*D*). A few genes of potential relevance to intestinal homeostasis were confirmed as up-regulated (Fig. 2*E* and *SI Appendix*, Fig. S4). Gene ontology analysis, based on cellular components of down-regulated genes from RNA-seq, indicated annotations were strongly associated with MHCII protein complex assembly (Fig. 2*F*). In addition, KEGG gene set enrichment analysis supports the down-regulated transcriptome as antigen processing and presentation encoding genes (Fig. 2*G*). We explored the extent of the *Zip14* deletion on MHCII expression in other immune organs. Surprisingly, qPCR of RNA from pooled lymph nodes, as well as spleens of *WT* and *KO* mice showed the MHCII genes assayed exhibited equal transcript levels for both genotypes (*SI Appendix*, Fig. S4). These data show that the differential in MHCII expression with global *Zip14* depletion is likely limited to the small intestine.

### Zip14 Deletion Reduces MHCII Abundance in Enterocytes.

**Fig. 3.**
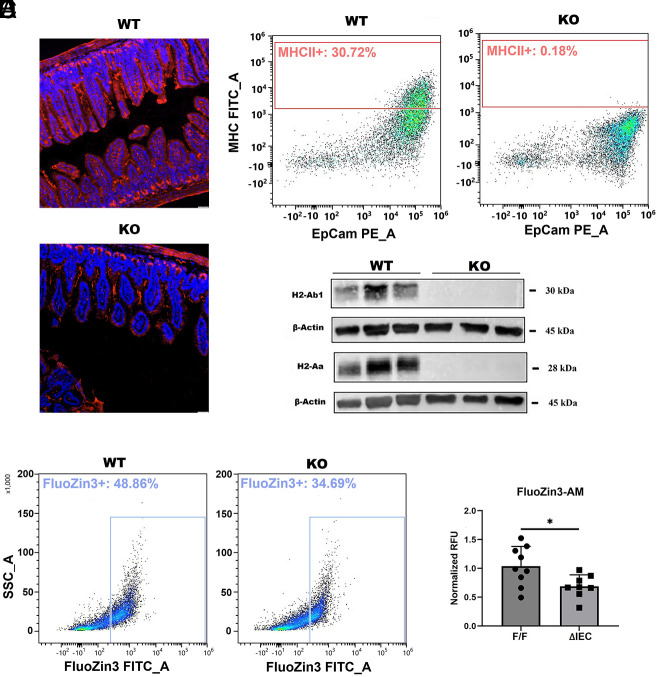
Confirmation of decreased MHCII expression in enterocytes and specific inflammation-related phenotypic changes produced from *Zip14* ablation. (*A*) Immunofluorescence staining for MHCII molecules in sections of intact small intestine from *WT* and *KO* mice. Staining is DAPI (blue) and MHCII (red). Bar is 50μm. (*B*) Frequency of MHCII in EpCAM+ cells from *WT* and *KO* mice using flow cytometry. (*C*) Western analysis of H2-AA and H2-AB1 expression in purified IECs from *WT* and *KO* mice (n = 3 mice/group). (*D*) Representative comparison of liable Zn2+ in purified IECs from *WT* and *KO* mice measured with FluoZin-3 fluorescent probe using flow cytometry. (*E*) Spectrofluorimetric analysis using FluoZin-3 with suspensions of organoids derived from male *Zip14*^*F/F*^ and *Zip14*^*ΔIEC*^ mice (n = 8 to 9 mice/group).

Using cross-sections of small intestine, images from immunofluorescence staining show that MHCII proteins are clearly visible along the apical surface of enterocytes of *WT* mice, but are minimally visible with comparable sections from *KO* mice (Fig. 3*A*). DAPI staining is comparable. In agreement, flow cytometry shows the markedly reduced abundance of MHCII proteins in EpCAM^+^ IECs from the *KO* mice compared to those from *WT* mice (Fig. 3*B*). Quantifiable differences in MHCII expression between the genotypes are 0.18% and 30.72%, respectively. Disruption of MHCII expression with the *Zip14* deletion was also shown at the protein level through western analysis of two key MHCII proteins, i.e., H2-Aa and H2-Ab1 (Fig. 3*C*). In support, a comparable reduction in both H2-Aa and H2-Ab1 was shown by immunohistochemistry of small intestine from *KO* mice (*SI Appendix*, Fig. S5). With respect to Zip14 function, as detected through flow cytometry using the fluorophore FluoZin-3, *Zip14* loss decreased the labile zinc abundance in EpCAM^+^ IECs by 14% (Fig. 3*D*). Similarly, spectrofluorimetric analysis of IECs in suspension, using the same fluorescent probe, showed a comparable reduction in labile zinc (Fig. 3*E*). The overall concentrations of elemental zinc in purified IECs did not change (*SI Appendix*, Fig. S6).

### ATAC Sequencing Reveals Chromatin Accessibility Influences MHCII Expression.

**Fig. 4.**
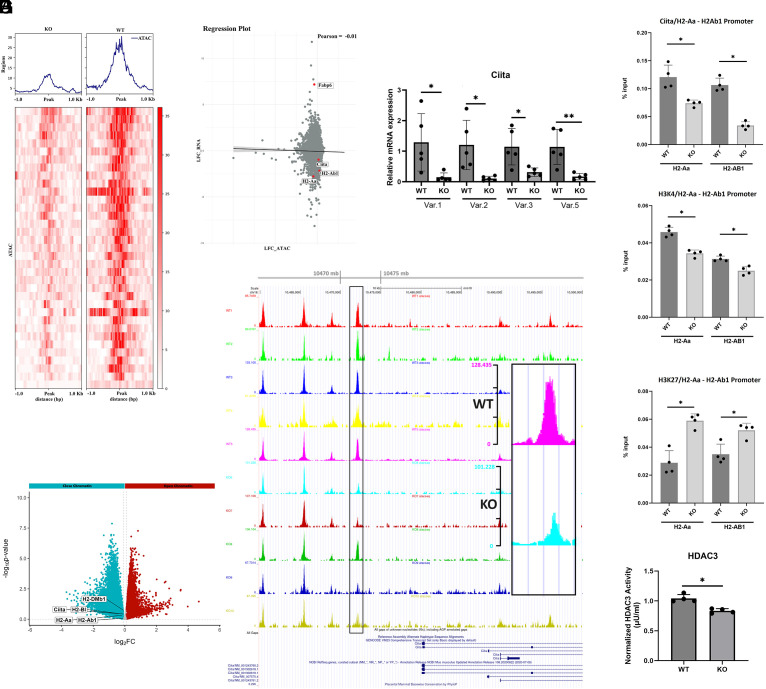
MHCII expression is differentially regulated by transcriptional changes through regulatory element occupancy produced with *Zip14* ablation as determined by ATAC-sequencing. (*A*) Heatmap of ATAC-seq. dataset showing lesser abundance of closed chromatin from purified IECs of *KO* mice vs. *WT* mice, with corresponding summary (*Top*) (n = 6 mice/group). (*B*) Volcano plot showing fold change (FC ± 2.0 to 8.0) of DEGs with increased (blue) and decreased (red) chromatin accessibility in specific regions based on ATAC-seq. Specific DEGs are identified. (*C*) Scatter plot showing the relationship of DEGs obtained through RNA-seq to chromatin accessibility assessed through ATAC-seq. Down-regulated genes of interest are identified. Up-regulated *Fabp6* is shown as a control. (*D*) Decreased abundance of *Ciita* transcripts in purified IECs from *Zip14 KO* mice shown through qPCR assays using four regulatory regions of the *Ciita* gene (n = 5 mice/group). (*E*) Peak densities in the regulatory region of Ciita highlight the reduction in open chromatin produced from the *Zip14* ablation. (*F*) ChIP assays showing decreased occupancy of Ciita and H3K4 for *H2-Aa* and *H2-Ab1* promoters and increased occupancy for H3K27 with the same promoters in purified IECs of *KO* mice compared to that of *WT* mice (n = 4 mice/group). (*G*) HDAC3 activity in nuclear extracts of IECs from *WT* and *KO* mice (n = 4 mice/group).

As a next step to explore the cause of the differential in MHCIIs, we used ATAC-sequencing to evaluate chromatin accessibility. PCA of the ATAC-seq dataset suggested that overall accessibility of the IECs was widely different between the two genotypes (*SI Appendix*, Fig. S7). The ATAC-seq data demonstrated a lower abundance of closed chromatin of the DEGs in the *Zip14 KO* mice, as shown with the heatmap in Fig. 4*A*. The abundance of open chromatin was correspondingly greater (*SI Appendix*, Fig. S8). The volcano plot derived from ATAC-seq separated significant peaks based on decreased or increased chromatin accessibility (Fig. 4*B*). Of note, specific MHCII genes, including *H2-Aa* and *H2-Ab1*, as well as *Ciita*, showed decreased chromatin accessibility. The relationship between expression of specific DEGs obtained through RNA-seq vs. chromatin accessibility based on ATAC-seq is shown in Fig. 4*C*. Downregulation of *Ciita* and specific *MHCII* transcripts were correlated with respect to decreased chromatin accessibility. Similarly, as a control, *Fabp6*, a highly up-regulated gene with *Zip14 KO* ablation, had the opposite correlation. Next, we wanted to more directly evaluate the effect of *Zip14 KO* ablation on Ciita expression. qPCR assay of four *Ciita* mRNA variants demonstrated a uniform downregulation in expression with *Zip14* deletion (Fig. 4*D*). Moreover, in support of *Ciita* downregulation, peak intensity of the first −7,905 kb and peak score of 13 (Chr16: 10,488,279 to 10,528,418) of the *Ciita* start site was less with DNA from IECs of the *Zip14 KO* mice compared to corresponding values for those from *WT* mice (Fig. 4*E*). By comparison, peak intensity at the start site of *Fabp6*, a gene which was confirmed by qPCR to be substantially up-regulated in IECs of the *KO* mice (Fig. 2*E*), was increased at −24,840 kb (TTS) with a peak score of 21.5 (Chr11: 43,596,049 to 43,601,540) (*SI Appendix*, Fig. S9).

We used chromatin immunoprecipitation (ChIP) assay of promoter regions of specific down-regulated MHCII genes, *H2-Aa* and *H2-Ab1*, to assess promoter occupancy by the Ciita transcription activator. With both genes, Ciita occupancy was less with chromatin derived from IECs of the *KO* mice, supporting a role of the Ciita transactivator in the downregulation of these MHCII genes (Fig. 4*F*). To further explore whether Ciita’s interaction with the *H2-Aa* and *H2-Ab1* gene promoters is functionally related to active chromatin, we used ChIP to quantify histone modification methylation sites in *KO* and *WT* IECs. Pull-down of H3K4me3, a marker of active chromatin, revealed reduced association near the Ciita interacting sites of *H2-Aa* and *H2-Ab1* with mouse *Zip14 KO* chromatin. Conversely, H3K27me3, a marker of repressive histone modification, showed increased association with *Zip14 KO* IEC chromatin surrounding the *H2-Aa* and *H2-Ab1* Ciita binding sites (Fig. 4*F*). These findings collectively support a direct role for Ciita in the altered transcriptional regulation of the *H2-Aa* and *H2-Ab1* genes in *Zip14* ablated intestinal epithelial cells. In support of these latter data, we measured activity of HDAC3, the prevalent HDAC in the intestine, and found activity was decreased in *KO* mice compared to the *WT* mice (Fig. 4*G*).

### Zinc Supplementation Reverses Epigenetically Depressed MHCII and Ciita Expression.

**Fig. 5.**
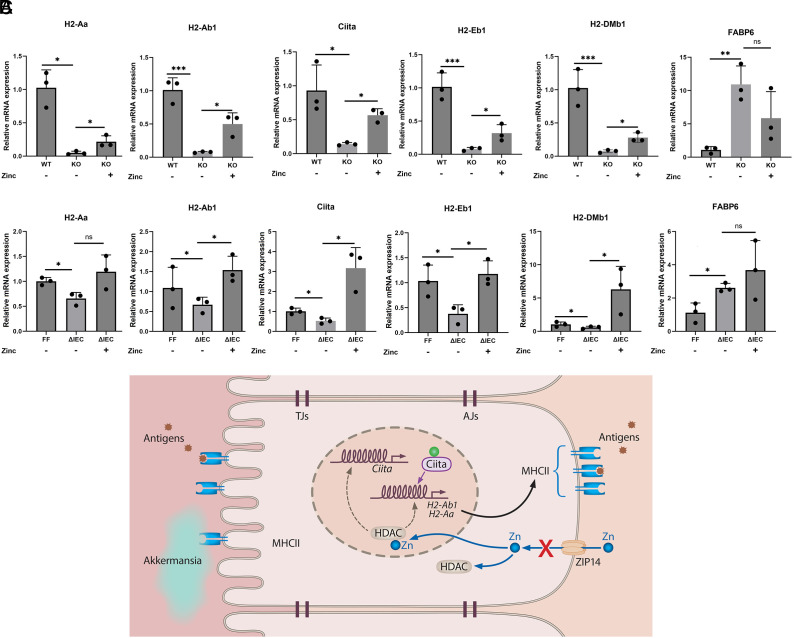
Zinc supplementation restores normal MHCII expression for mice and intestinal organoids with *Zip14* deletion. (*A*) Zinc supplementation of the mice was provided as 6.7 mM zinc in the drinking water for 7 d (n = 3 mice/group). (*B*) Zinc was added to the culture medium at 15 μM for intestinal organoids produced from *Zip14^F/F^* and *Zip14*^*ΔIEC*^ mice for the last seven days of the 10-d culture period (n M for intestinal organoids produced from *Zip14^F/F^* and *Zip14*^*ΔIEC*^ mice for the last seven days of the 10-d culture period (n = 3 organoid cultures from separate mice/group). ∗P<0.05, ∗∗P<0.01, and ∗∗∗P<0.001. (*C*) Model linking depressed MHCII expression in enterocytes with Zip14-mediated Zn transport. Deletion of *Zip14* (indicated by the X) produces inflammation of the small intestine, dysbiosis with Akkermansia overgrowth, and epigenetic HDAC-dependent modifications to chromatin, leading to an atypical transcriptome profile. Ciita and MHCII expression, including H2-Aa and H2-Ab1, are depressed thus altering intestinal homeostasis.

We next explored the possibility that the deficit in cellular zinc produced with *Zip14* ablation could be restored by supplementation with zinc. Supplementing the drinking water of the *WT* and *KO* mice with zinc (7.6 mM) for 7 d reversed the repressed *MHCII* and *Ciita* gene expression in IECs from the *KO* mice to levels close to those of the *WT* mice (Fig. 5*A*). In contrast, expression of *Fabp6*, markedly elevated in the *KO* mice, was reduced with supplemental zinc, thus serving as a positive control.

To examine *MHCII* expression from another perspective, we used organoids from mice of *Zip14^F/F^* and *Zip14*^*ΔIEC*^ mice. Capitalizing on our previous evidence that these organoids remain viable for at least 10 d in culture, and importantly retain the biochemical phenotype associated with *Zip14* deficiency (10), we evaluated the response to zinc (added in vitro at 15 μM) in exploring normal *MHCII* expression. Clearly, the depressed *MHCII* and *Ciita* expression produced with the *Zip14*^*ΔIEC*^ was reversed with supplemental zinc in vitro (Fig. 5*B*). Here, *Fabp6* expression, serving as a positive control, was not normalized in zinc-treated organoids with the *Zip14* deletion (Fig. 5*B*).

Taken together, we propose these results show that proper zinc transport via Zip14 is necessary for the synthesis of specific MHCII molecules by IECs and that when *Zip14* is deleted or dysfunctional, it is accompanied by indices of intestinal inflammation. Moreover, it is proposed that zinc-dependent HDAC epigenetic mechanisms, acting through Ciita, influence these key factors required for intestinal immune defense.

## Discussion

Among the effects of *Zip14* deletion on phenotype is chronic low-grade intestinal inflammation, characterized by increased permeability and endotoxemia (9, 24, 25). As shown here with mice at steady state, a marked increase in Il-6 expression by IECs, and increased fecal calprotectin and lipocalin-2, two markers of intestinal inflammation (26), support the inflammatory phenotype of Zip14 deficiency. Moreover, the elevated fecal IgG/IgA levels of the *Zip14* deficient mice are consistent with an activated immune response (27, 28). These characteristics of *Zip14* deletion are in common with those of inflammatory bowel disease (29).

Our previous experiments have demonstrated that Zip14 is most highly expressed in enterocytes of the proximal small intestine and diminishes progressively in distal regions of the gastrointestinal tract (9). Zip14 is localized primarily at the basolateral membrane of enterocytes. This orientation suggests that Zip14 transports circulating endogenous systemic zinc into enterocytes for functional roles, rather than as a component of the transepithelial absorptive pathway. This cellular orientation is consistent with the demonstrated transfer of systemic zinc and manganese for excretion into the intestinal lumen (30, 31). Stoichiometry of circulating zinc, manganese, and iron concentrations, with their abilities to easily exchange with ligands and substrate affinity, favor zinc as the main physiologic substrate of Zip14 (32). We have detected all of the Zip and ZnT transporters in enterocytes at the transcript level (10). With the exception of Zip4, which is needed for transepithelial transport for dietary zinc absorption (3, 4), the roles of the other zinc transporters in gastrointestinal homeostasis are not well understood.

The role of MHCII in intestinal defense has received considerable attention recently (33–38). MHCII molecules in the proximal small intestine are abundant in enterocytes and intestinal stem cells but are only minimally expressed in tuft, goblet, Paneth, and enterochromaffin cells (37). MHCII production in the small intestine tends to be constitutive (33), unless perturbed through dysbiosis or inflammation. Intestinal *MHCII* expression is depressed with aging and when a high fat diet is consumed, however (35, 36). As demonstrated here with highly purified IECs from the proximal small intestine, the production of MHCII molecules is also greatly influenced by *Zip14* ablation. The responsiveness of *MHCII* expression in IECs with *Zip14* ablation adds a new dimension to a role for Zip14-mediated zinc transport in maintaining intestinal homeostasis.

Commensal microbiota of the gastrointestinal tract influence intestinal MHCII. Specifically, intestinal *MHCII* expression in mice is responsive to specific microbiota and circadian rhythms (34, 38). The mice used for our experiments were all killed at the same time of the day, minimizing temporal effects. Food allergens have been reported to induce MHCII in IECs through a Ciita-dependent mechanism (37). Mice used in our experiments were fed the same natural-component diet since weaning. This minimizes the possibility that dietary allergens produced the differential in MHCII molecules reported here. In that regard, it is relevant to emphasize that the greatest expression of *Zip14* is in the proximal small intestine (9), where food components and allergens are first encountered.

The very significant abundance of *Akkermansia*, a gram-negative anaerobic, mucus-degrading bacterium, in feces of the *KO* mice is of considerable interest. This finding confirms our previous identification of *Akkermansia* as more abundant in feces from mice with enterocyte-specific *Zip14* ablation (10). It is also in agreement with the histological evidence that the *Zip14*^*ΔIEC*^ mice have less alcian blue stained luminal mucus than controls (10). *Akkermansia*, as a commensal gut microorganism, is widely viewed as anti-inflammatory, a probiotic, and beneficial to host health through improvement in intestinal barrier and stimulation of epithelial proliferation (39, 40). In contrast, excessive abundance of *Akkermansia* in the intestine may be damaging to the intestinal barrier, particularly when there are proinflammatory conditions present (41). Therefore, the overabundance of *Akkermansia* with loss of Zip14 transport activity could be beneficial or deleterious for the host. The teleological basis for this shift in species of commensal flora with *Zip14* deletion has not been established. Zip14 deficiency may represent a model to evaluate this microbial/intestinal interaction.

IECs interact with other immune cells, including T cells in the lamina propria and intraepithelial T cells (24, 33, 38, 42). Here, we have shown that T cells produce Zip14, but surprisingly global *Zip14* ablation does not alter T cell *MHCII* expression. Of note, Zip14 is inducible by LPS in human macrophages (43). Zinc trafficking in T cells has focused primarily on Zip6 (44, 45), which influences MHCII expression (44). It has been proposed that zinc transporters are involved in many aspects of immune function (46).

Regulation of *MHCII* gene expression, and the role played by Ciita, is complex, involving binding with numerous transcription factors, and is known to be influenced by epigenetic histone modifications (47–50). Interestingly, it has been shown that intestinal microbiota composition alters HDAC3 activity and MHCII expression (34). As shown here with the ATAC-seq, *Zip14* ablation alters chromatin accessibility (Fig. 4*A*). Histone modifications have a profound influence on chromatin accessibility. Using chromatin from *WT* and *KO* mice for ChIP assays, the H3K4 and H3K27 histone marks for active (open) and inactive (closed) chromatin, respectively, show that promoters for *H2-Aa* and *H2-Ab1*, two key MHCII components, exhibit expected reflections of chromatin accessibility. The balance of HAT (histone acetyltransferase) and HDAC (histone deacetylase) activity at specific loci helps to control accessibility. Specific classes of HDACs and HATs are zinc-dependent enzymes that control epigenetic modifications (51). It has been postulated that zinc dependency of these metalloenzymes makes them targets of zinc deficiency including that produced through atypical zinc transport (51, 52). In agreement with those suggestions, as demonstrated here, zinc supplementation of mice in vivo and *Zip14*^*ΔIEC*^ intestinal organoids in vitro restores *MHCII* and *Ciita* expression.

Collectively, our results are consistent with a model where chromatin occupancy patterns in enterocytes, produced by Zip14 deficiency, create a zinc-related HDAC/HAT imbalance that alters chromatin accessibility for regulatory factors and hence the global transcriptome profile, importantly including depressed MHCII expression (Fig. 5*C*). We have capitalized on the emerging recognition of the role enterocytes can play in MHCII-dependent intestinal defense and showed that some epigenetic modifications are sensitive to zinc availability through altered transport. These experiments add functional metal transport to an important aspect of intestinal homeostasis and immune defense.

## Materials and Methods

Detailed methods are available in the *SI Appendix*.

### Mice and Husbandry.

The *Zip14* knockout mouse strains used in these studies have been described in detail previously (10, 25). Briefly, targeted deletion of introns 4 to 8 of *Zip14* were used to produce both the global and enterocyte-specific knockouts. The global knockout strain is designated as *KO* and *WT* as the control. Mice with the enterocyte-specific *Zip14* deletion were designated *Zip14*^*ΔIEC*^ while controls were designated *Zip14^F/F^*. The mice were not fasted prior to being killed which was always performed between 11 AM and 1 PM. Mice were euthanized by exsanguination via cardiac puncture while under isoflurane anesthesia. Protocols were approved by the University of Florida Institutional Animal Care and Use Committee (No. 202007015).

### Isolation of Intestinal Epithelial Cells.

The IECs were isolated as described in detail earlier (10). Briefly, the first 20 cm of small intestine (duodenum and upper jejunum) was excised from mice and the lumen was individually perfused with ice-cold buffer (10 mM EDTA; 10 mM HEPES; 0.9% NaCl). IECs were liberated from underlying tissue by repeated vortexing with EDTA-containing phosphate buffer (DPBS). The final cell suspension was centrifuged, and the cells were prepared for assays or other downstream applications.

### Fluorescence-Activated Cell Sorting.

The cells were stained with APC anti-mouse CD45 (Blood cells), FITC anti-mouse CD31 (Vascular cells), PE/Cyanine7 anti-mouse TER-119 (Lymphocytes), PE anti-mouse CD326 (EpCAM) (Epithelial cells), then resuspended in MACS buffer and used for FACS (53).

### Preparation and Culture of Small Intestinal Organoids.

The first 20 cm of proximal small intestines from both *Zip14^F/F^* and *Zip14*^Δ^*^IEC^* mice were excised and perfused with PBS, cut into 5 mm strips, split open, and placed in cell dissociation reagent (StemCell Technologies, Inc.). Cell domes were placed in Matrigel (Corning) and organoids were maintained for up to 10 d as described in detail earlier (10).

### RNA-Sequencing.

The isolated IECs were homogenized using a bullet blender. RNA was extracted and sequenced on an Illumina NovaSeq (2 × 100 bp) to achieve a minimum of 40 million reads per sample at the University of Florida NextGen DNA Sequencing Core Facility. Files were downloaded to the University of Florida HiPerGator computing cluster.

### ATAC-Sequencing.

Intestinal epithelial cells were isolated as above and were further purified for flow cytometry and cell sorting (53). The preserved IECs were sent to MedGenome (Foster City, CA). ATAC sequencing was performed as 75 bp paired-end reads on an Illumina HiSeq 2000.

### Quantitative PCR.

Total RNA was extracted from IECs, intestinal organoids, spleen, and lymph nodes and assessed for quality and concentration spectrophotometrically. Quantitative PCR (qPCR) was performed with detection using either EXPRESS SYBR GreenER Supermix with ROX or specific TaqMan assays. Primer/probes sequences used have been published (10, 26) or are listed in the *SI Appendix*. *Gapdh* RNA or *18S* rRNA were used as normalizers with relative expression measured by the *2-ΔΔCt* method.

### Western Analyses.

IECs were lysed and SDS-PAGE was used to separate the lysate proteins, followed by transfer to nitrocellulose membranes. After incubations with primary and secondary antibodies, separated proteins were detected by chemiluminescence.

### Chromatin Immunoprecipitation Assays.

Isolated IECs were suspended in PBS. Precision sonication of chromatin produced a nuclear extract with fragments between 200 and 700 bp (10). Specific antibodies against Ciita, anti-Histone H3 (tri-methyl K4), or anti-trimethyl-Histone H3 (Lys27) were used for individual ChIP assays by qPCR.

### Microbiome Analysis and ELISA Assays.

Fecal IgA, IgG, calprotectin, and lipocalin-2 concentrations were measured by ELISA. Characterization of fecal microbial taxa involved DNA extraction from each fecal sample and 16S rRNA sequencing (CosmosID, Germantown, MD), but data were analyzed in-house.

### Other Assay Methods.

Immunofluorescence staining with DAPI (nuclei) and MHCII antibody was performed on sections of intestinal tissue from *WT* and *KO* mice. HDAC3 activity of IEC nuclear extracts was measured spectrofluorimetrically. Labile intracellular zinc was measured by incubating fresh IECs with FluoZin-3 followed by analysis using flow cytometry or spectrofluorimetrically. Elemental analyses for zinc and manganese were by microwave plasma atomic emission spectroscopy (10). Sections of intestinal tissue of *WT* and *KO* mice were stained with hematoxylin (nuclei) and H2-Aa and H2-Ab1 primary antibodies, followed by secondary to visualize the immunohistochemistry.

### Statistical Analysis.

Data are presented as means ± SE of biological replicates (n). One organoid culture derived from cells of one individual mouse is an n=1. All experiments were replicated at least once for those using mice and multiple times for those using isolated cells, organoids, and extracts. GraphPad Prism 10.0 software was used for most analyses. Student’s *t* test was used to compare mice or organoids of two genotypes and one-way ANOVA was used for multiple comparisons. The Wilcoxon rank test was used for assays of fecal parameters. Statistical probabilities are indicated for each measured parameter, with P<0.05 or less considered statistically significant.

### Bioinformatics.

Sequencing data and downstream analyses were performed at the University of Florida Bioinformatics Core. Details are presented in the *SI Appendix*.

## Supplementary Material

Appendix 01 (PDF)

## Data Availability

RNA-seq and ATAC-seq datasets are deposited with the GEO database under accession number GSE280523 at National Center for Biotechnology Information Gene Expression Omnibus (54). All other data are included in the manuscript and/or *SI Appendix*.
