# Supplementary material for: Deletion of metal transporter Zip14 reduces major histocompatibility complex II expression in murine small intestinal epithelial cells
Source: Proc Natl Acad Sci U S A. 2024 Dec 30;122(1):e2422321121. doi: 10.1073/pnas.2422321121 (PMC11725848; doi:10.1073/pnas.2422321121)
Supplement: Supplementary file 1 — Appendix 01 (PDF) [file pnas.2422321121.sapp.pdf]

Supporting Information for

Deletion of Metal Transporter Zip14 (Slc39a14) Reduces Major Histocompatibility Complex II Expression in Murine Small Intestinal Epithelial Cells

Felix R. Jimenez-Rondan[a], Courtney H. Ruggiero[a], Fahong Yu[b], Alberto Riva[b], Lauren S. Stafford[c], Tyler R. Cross[c], Joseph Larkin III[c], and Robert J. Cousins[a,b] [1]

[a]Center for Nutritional Sciences, Food Science and Human Nutrition Department, College of Agricultural and Life Sciences, University of Florida, Gainesville, FL 32611; [b]Interdisciplinary Center for Biotechnology Research, University of Florida, Gainesville, FL 32611; [c]Department of Microbiology and Cell Science, College of Agricultural and Life Sciences, University of Florida, Gainesville, FL 32611; [d]Department of Biochemistry and Molecular Biology, College of Medicine, University of Florida, Gainesville, FL 32611; [1]Corresponding author: Robert J. Cousins, Food Science and Human Nutrition Department, University of Florida, Gainesville, FL 32611; Phone: 352-538-0814; cousins@ufl.edu.

Corresponding Author: Robert J. Cousins.
E-mail: cousins@ufl.edu

This PDF file includes:

Figs. S1 to S9
Tables S1 to S2
SI References

This is the footnote text that appears at the bottom of the page.

Materials and Methods

Mice and Husbandry. The Zip14 knockout mouse strains used in these studies have been described in detail previously (1, 2). Targeted deletion of introns 4-8 of Zip14 were used to produce both the global and enterocyte-specific knockouts. For the global knockout strain, Het versus Het mice were crossed to produce the Zip14+/+ and Zip14-/- strains, subsequently designated as WT and KO, respectively. Mice with the enterocyte-specific Zip14 deletion were designated $Zip14^{\Delta IEC}$ while controls were founder $Zip14^{F/F}$ mice. Backcrosses for both strains were to C57BL/6 mice. Generations for both strains were in excess of F15. Unless specifically indicated otherwise, experiments were conducted with female mice of this *Zip14* global *KO* strain. The mice were used as young adults (8-16 weeks of age). Maintenance consisted of shoebox caging, a 12/12 light/dark cycle and ad libitum access to diet (Harlan 7012) and tap water. In one experimental series, drinking water was supplemented with zinc at 7.6 mM as $ZnSO_4$ (3). For fecal collections, mice were maintained in individual hanging wire cages. The mice were not fasted prior to euthanasia, which was always performed between 11 AM and 1 PM. Mice were euthanized by exsanguination via cardiac puncture while under isoflurane anesthesia. Tissues excised from anesthetized individual mice were small intestine, spleen and lymph nodes (pooled axillary, inguinal, brachial, mesenteric and superficial cervical) (4). Protocols were approved by the University of Florida Institutional Animal Care and Use Committee (No. 202007015).

Isolation of Intestinal Epithelial Cells (IECs). The cells were isolated as described in detail earlier (2). The first 20 cm of small intestine (duodenum and upper jejunum) was excised from mice and the lumen was individually perfused with ice-cold buffer (10 mM EDTA; 10 mM HEPES; 0.9% NaCl). IECs were liberated from underlying tissue by repeated vortexing, using an EDTA-containing phosphate buffer (DPBS) and subsequent centrifugations. Cell pellets were washed with cold DPBS to remove any residual EDTA buffer, and TrypLE Express Enzyme was added to dissociate cell aggregates. The cells were gently resuspended using a pipette and incubated at room temperature for 10 min, with periodic trituration. The cell suspension was passed through a 70 μm cell strainer into cold DPBS. Flow-through solution containing the single-cell suspension was centrifuged to pellet the cells which were treated with cell removal medium (Akadeum, cat. 11510-211), suspended in Live/Dead staining solution and transferred to FACS tubes. The final cell suspension was centrifuged, and some of the cells were prepared for staining and cell sorting, while cells designated for RNA-Seq were placed in cryopreservation medium (NutriFreez® D10, Fisher Scientific, cat. 05-713-1E). Cells used for RNA and protein isolations were lysed in Trysol or RIPA Buffer, respectively, or were stored at -80°C until processed for ATAC-Sequencing.

Fluorescence-Activated Cell Sorting (FACS). The cells were stained with antibodies for specific cell surface markers, APC anti-mouse CD45 (Blood cells), FITC anti-mouse CD31 (Vascular cells), PE/Cyanine7 anti-mouse TER-119 (Lymphocytes), PE anti-mouse CD326 (EpCAM) (Epithelial cells), followed by washing to remove unbound antibodies. Finally, the stained cells were resuspended in MACS buffer and transferred to FACS tubes mounted with a 35 μm cell strainer cap to remove cell aggregates. The FACS protocol followed that of Ge et al. (5). A Sony SH800 and Cytoflex STR cell sorter was used. The data obtained were analyzed using FlowJo software. Following acquisition, cells were mixed with AOPI staining solution for counting with Auto 2000. For ATAC-seq, cells were placed in cryopreservation medium as above.

Preparation and Culture in Intestinal Organoids. The first 20 cm of proximal small intestines from both $Zip14^{F/F}$ and $Zip14^{\Delta IEC}$ mice were excised and perfused with PBS, cut into 5 mm strips, split open and placed in cell dissociation reagent (StemCell Technologies, Inc.). Cell domes were placed in Matrigel (Corning) and organoids were placed into wells of 6-well plates as described in detail earlier (2). The organoids were cultured for 10 days in IntestiCult medium (StemCell), which was replaced every 3 days. For some organoid cultures, zinc-containing medium (as $ZnSO_4$; 15 µM), was used starting at day 3 of the 10-day culture period.

RNA-Sequencing. The IECs were placed in RNase-free tubes containing TRIzol and RNase-free zirconium oxide beads (1.0 mm diameter, NextAdvance, Troy, NY, USA). The cells were homogenized using a bullet blender, and the RNA was purified using a modified RNeasy Mini RNA extraction kit (Qiagen, Germantown, MD, USA). The RNA was treated with DNase, and its integrity was confirmed with an Agilent Bioanalyzer, ensuring all samples had an RNA integrity number above 8.0. Poly-A selected RNA-seq libraries were prepared using the Illumina mRNA Prep kit (Illumina, San Diego, CA, USA), pooled to equal molarity, and sequenced on an Illumina NovaSeq (2 x 100 bp) to achieve a minimum of 40 million reads per sample at the University of Florida NextGen DNA Sequencing Core Facility. The resulting FastQ files were downloaded to the University of Florida HiPerGator computing cluster. Raw FastQ files, counts, and normalized counts per million data were uploaded to GEO: ...

ATAC-Sequencing. IECs were isolated as above and sorted further using flow cytometry (4). The preserved IECs were sent to MedGenome, Foster City, CA, USA for library preparation and Assay for Transposase-Accessible Chromatin (ATAC) sequencing. Briefly, nuclei were isolated from the IECs and incubated with pre-loaded Tn5 transposase (Illumina, cat. FC-121-1030) and sequencing adapters. The assembled protein A-Tn5 adapter transposome was activated by Mg^{2+} to generate adapter-flanked DNA fragments cut near the proteins of interest. The transposed DNA was then extracted, and PCR amplified using NEBNext High-Fidelity 2X PCR Master Mix (New England Biolabs, cat. M0541S). The library was purified with AMPure XP Magnetic Beads (Beckman Coulter, cat. A63880), and its quality was assessed using a BioAnalyzer High-Sensitivity DNA Analysis kit (Agilent, cat. 5067–4626). Sequencing was performed as 75 bp paired-end reads on an Illumina HiSeq 2000.

Quantitative PCR. Total RNA was extracted from IECs and organoids as above and was assessed for quality and concentration spectrophotometrically. Then first strand cDNA was produced from the RNA using High-Capacity RNA-to-DNA reagents (Applied Biosystems). Samples were diluted with DNase/RNase-free water and real-time PCR was performed with a QuantStudio 3 qPCR sequence detector (ThermoFisher). Detection was with either EXPRESS SYBR GreenER Supermix with ROX or specific TaqMan assays used as recommended (ThermoFisher). The primer/probes sequences used have been published (2, 6) or are listed below. q-PCR reactions were run in duplicate for each sample. All data presented were derived with different RNA preparations run at least twice. Gapdh RNA or 18S rRNA were used as normalizers with relative expression measured by the 2-ΔΔCt method.

Western Analyses. IECs were homogenized using the Bullet Blender with stainless steel beads in radioimmunoprecipitation buffer (RIPA) with protease/phosphatase inhibitors as described previously (2). Protein concentrations were determined using the Pierce BCA Protein Assay. Lysates were separated by SDS-PAGE on mini PROTEAN TGX gels (BioRad) and transferred to nitrocellulose membranes (Amersham) at 60V for 1 hour. Ponceau Red staining verified transfer uniformity, and membranes were cut into strips based on marker protein standards. Strips were washed three times with Tris-buffered saline-Tween 20 (TBST), rocking for 5 minutes at room temperature, blocked with BioRad Every Blot Blocking Buffer for 5 minutes, and incubated with primary antibodies overnight at 4°C with rocking. Strips were washed three times with Tris-buffered saline-Tween 20 (TBST), rocking for 5 minutes at room temperature, before and after incubation of secondary antibody, which was for 1-1.5 hour at room temperature with rocking. Chemiluminescence was detected using SuperSignal West Pico Plus (ThermoFisher) and visualized using Protein Simple FluorChemE.

Chromatin Immunoprecipitation (ChIP) Assays. IECs were suspended in PBS and crosslinked with 1% formaldehyde for 10 minutes at room temperature, followed by quenching with glycine for 5 minutes. Cells were lysed in ChIP sonication lysis buffer with protease inhibitors (Cell Signaling, cat. 7012) and sonicated using a Bioruptor (Diagenode) to produce chromatin fragments between 200 and 700 bp. Immunoprecipitations were performed overnight at 4°C with Ciita, anti-Histone H3 (tri-methyl K4), or anti-trimethyl-Histone H3 (Lys27) antibodies. Protein G magnetic beads were added, and chromatin was eluted with 1X ChIP elution buffer (Cell Signaling, cat. 7009), followed by cross-link reversal with 5M NaCl and Proteinase K. DNA was purified using spin columns and stored at -20°C for further analysis (2, 6).

Microbiome Analysis and ELISA Assays. Fecal samples were collected and stored at -80°C. After thawing, samples were weighed then homogenized in cold PBS with protease inhibitors to equal concentrations. Protein concentrations of fecal lysates were determined using the Pierce BCA Protein Assay. IgA, IgG, calprotectin, and lipocalin-2 were quantified by ELISA (Fortis, Abcam, R&D systems) according to the manufacturers' protocols. Microbial taxa were identified by *16S* rRNA sequencing (CosmosID).

Other Assay Methods. For immunofluorescence, duodenal tissues from *WT* and *KO* mice were collected, washed with cold PBS, fixed with 10% formalin for 24 h at room temperature, and embedded in paraffin. Sections were cut to 5 μm thickness and mounted. Sections were developed with rabbit MHCII primary antibody (1:100), followed by the addition of an IgG Alexa 594 conjugate antibody (Molecular Probes, Eugene, OR). Nuclear counterstaining was performed with 4,6-diamidino-2-phenylindole (DAPI) (Invitrogen). Samples were imaged with an Olympus VS200 whole-slide scanner. Nuclear extractions of IECs, using EpiQuick Nuclear Extraction Kit (Epigentek, cat. OP-0002), were used for measuring the HDAC3 activity (BioVision, cat. K331-100) via SpectraMax M5 spectophotometer (Molecular Devices). Labile intracellular zinc levels in IECs were assessed using FluoZin-3 with flow cytometry (FC) and spectrofluorimetry (2, 7). MHCII molecule expression on IECs was also determined using FC with labeled MHCII and EpCAM antibodies (5, 8). Zinc and manganese concentrations were measured by plasma microwave atomic emission spectroscopy (Agilent 4210). The immunohistochemistry was carried out on 5 μm sections of intestine tissue, incubated with rabbit H2-Aa (1:200) and H2-Ab1 (1:200), followed by hematoxylin (Vector, Burlingame, CA) for visualization by light microscopy. Samples were imaged with an Olympus VS200 whole-slide scanner. Images were produced at the Pathology core of the University of Florida.

Statistical Analysis. All data are presented as means ± SE of biological replicates. Organoid cultures from individual mice were considered as n=1. Statistical analyses were performed using GraphPad Prism 10. Student's t-test was used for two-genotype comparisons, and one-way ANOVA was applied for multiple comparisons. Wilcoxon rank tests were employed for fecal parameter analyses. P-values < 0.05 considered statistically significant.

Bioinformatics. 16S rRNA sequencing data and downstream analyses were performed at the University of Florida Bioinformatics Core.

Antibodies. The following antibodies were used for flow cytometry: APC anti-mouse CD45 (BioLegend, cat. 103112), FITC anti-mouse CD31 (BioLegend, cat. 102406), PE/Cyanine7 anti-mouse TER-119 (BioLegend, cat. 116222), and PE anti-mouse CD326 (EpCAM) (Thermo Fisher, cat. 12-5791-83). For immunofluorescence: MHC Class II (I-A/I-E) (Thermo Fisher, cat. 14-5321-82). Western blot antibodies included Zip14, MHC Class II (H2-Aa) (Thermo Fisher, cat. PA5-116820), and anti-H2-Ab1 (MyBioSource, cat. MBS9143467).

Primers/Probes. The primers and probes used in the present study were designed by OriGene: EpCam (MP217790), Cd31 (MP211049), Il6 (MP206798), Zip14 (2,6), Crb3 (MP202987), Fabp6 (MP204656), Cfi (MP202883), H2-DMb1 (MP206139), H2-Aa (MP206133), H2-Ab1 (MP206134), Cd74 (MP201948), and H2-Eb1 (MP206136). The *Ciita* primers were designed using NCBI Primer3 software:

Ciita-205: 5'-TGCAGGCGACCAGGAGAGACA-3', 5'-GAAGCTGGGCACCTCAAAGAT-3';

Ciita-206: 5'-GAGACGCTCAACTTGTCCCA-3', 5'-AGGCTCTTGGCTCCTTTGTC-3';

Ciita-201: 5'-CAAGCTGTTGCAGGACATGG-3', 5'-CCTCTGCTCCAATGTGCTCT-3'; and

Ciita-203: 5'-GGGAGGCCTATGCCAACATT-3', 5'-CAAAGCCTTCCTCTGCTCCA-3'.

For the ChIP assay, the primers were:

chip_mH2-Aa_F: 5'-AGGTGGATCATCTCACAATTTGG-3',

chip_mH2-Aa_R: 5'-GCTTGCATGCATCATGAGTTAGC-3';

chip_mH2-Ab1_F: 5'-AGGCAGAGGCTGCAGATTATTG-3', and

chip_mH2-Ab1_R: 5'-AGCAGACAAACATGGCCATTC-3'.

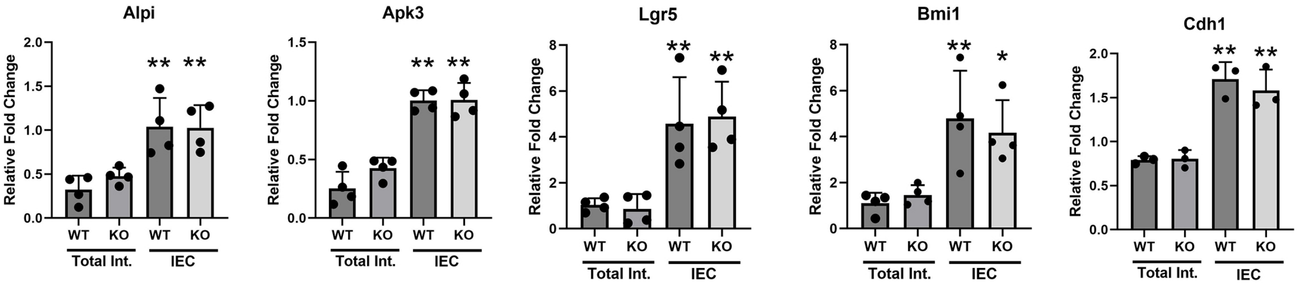

Fig. S1. qPCR of enterocyte-specific transcripts in WT and KO purified IECs compared to total intestine. ANOVA $^{*}P < 0.05$, $^{**}P < 0.01$

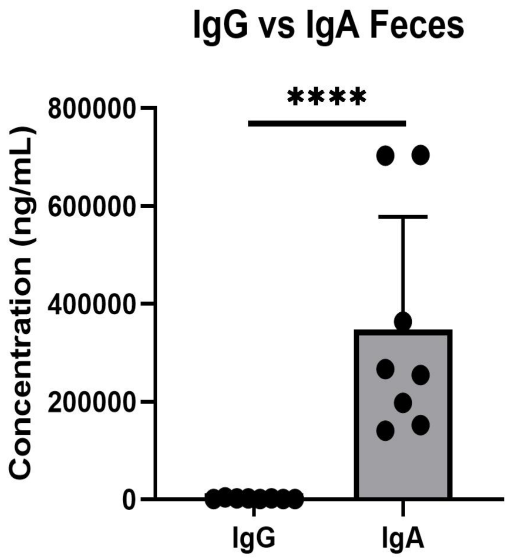

Fig. S2. Ratio of IgG to IgA concentrations from combined WT and KO mice feces.

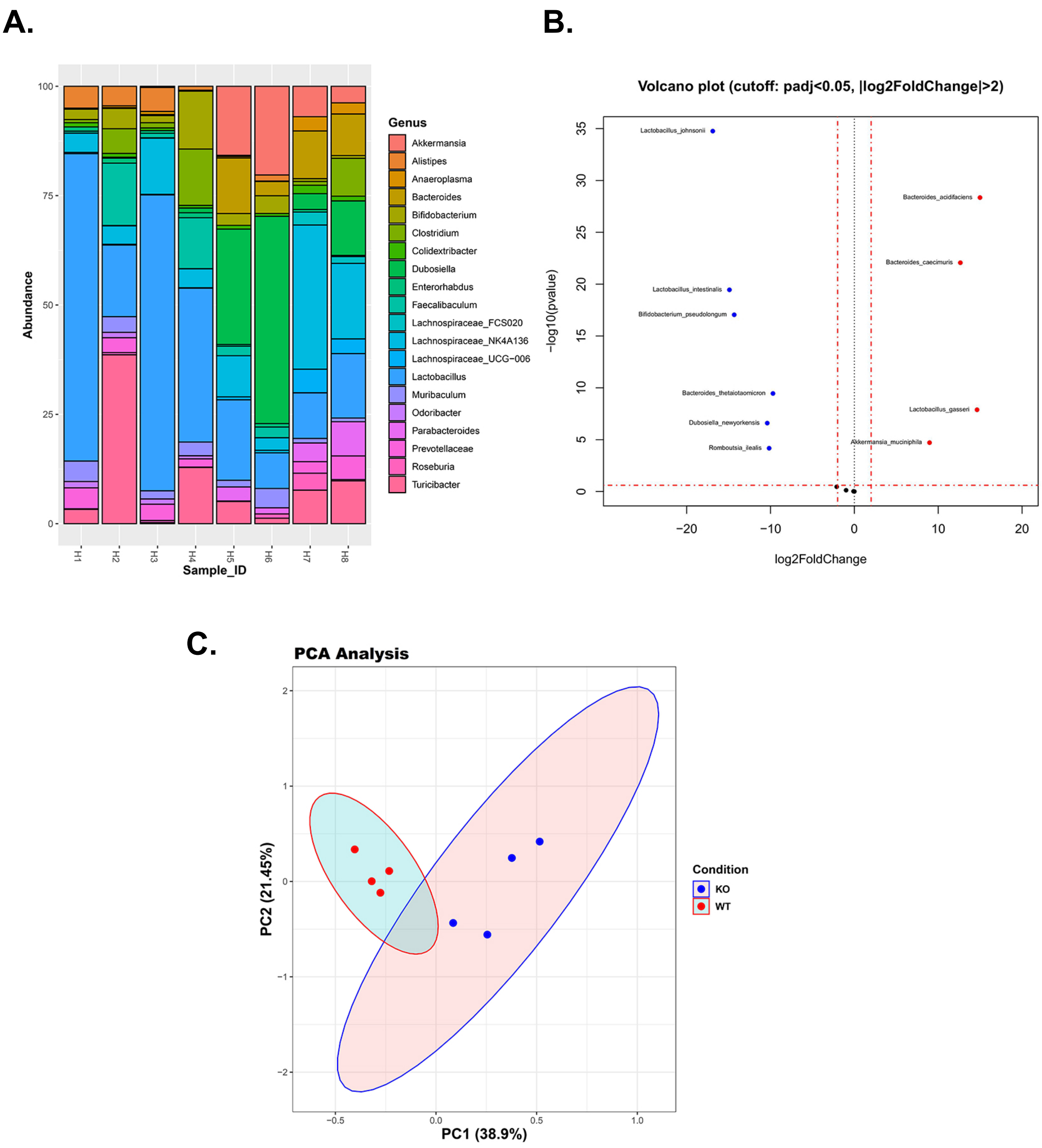

Fig. S3. A. Plot of $16S$-rRNA sequencing of microbiota taxa from feces of WT and KO mice. B. Volcano plot. C. Principal component analysis.

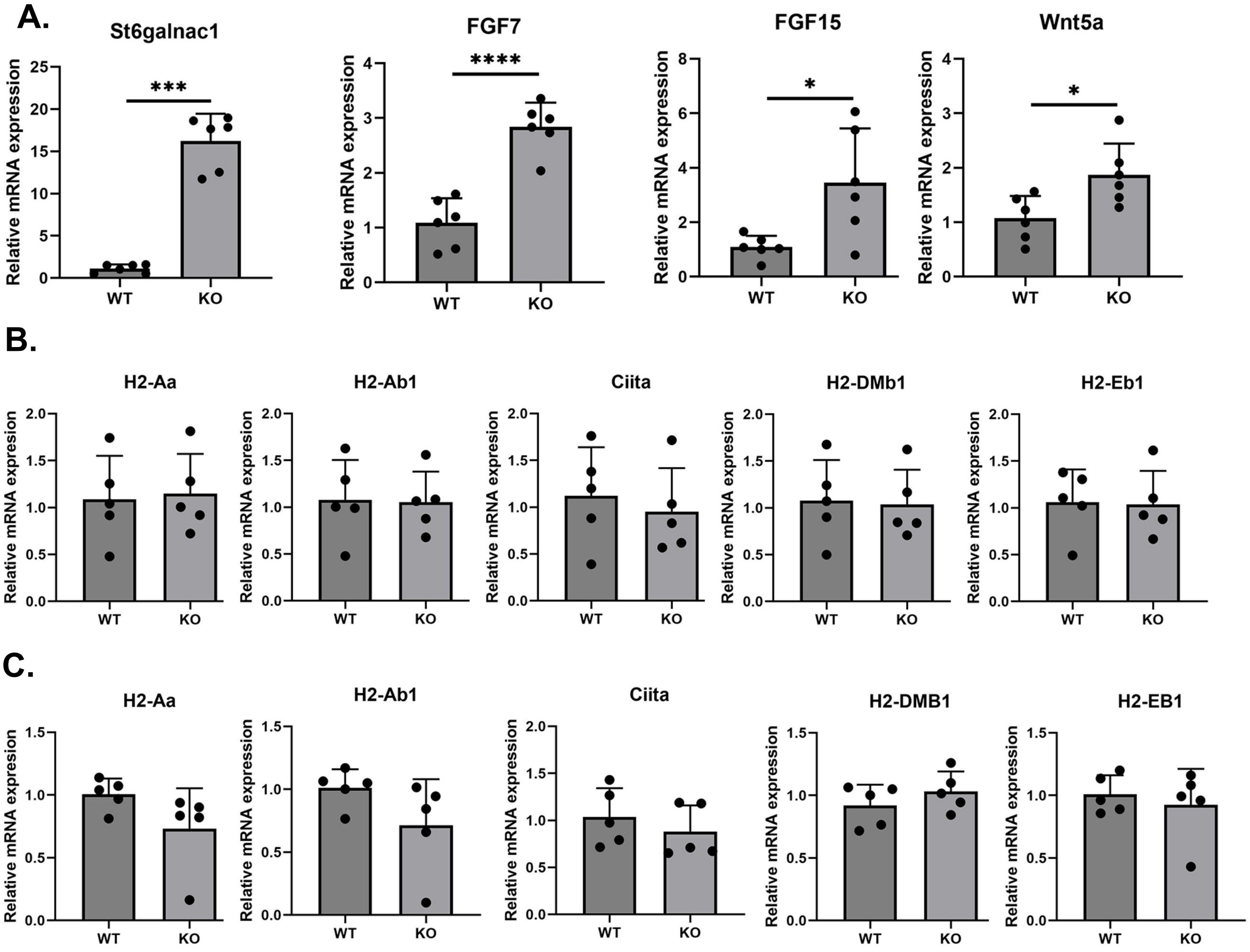

Fig. S4. Confirmation by qPCR assay of transcripts. A. Some upregulated genes obtained by RNA-seq relevant to intestinal homeostasis that are not shown in Fig.1B with RNA from purified IECs of small intestine from WT and KO mice. B. MHCII genes from spleen of WT and KO mice. C. MHCII genes from lymph nodes of WT and KO mice. $^{*}P < 0.05$, $^{***}P < 0.001$, $^{****}P < 0.0001$

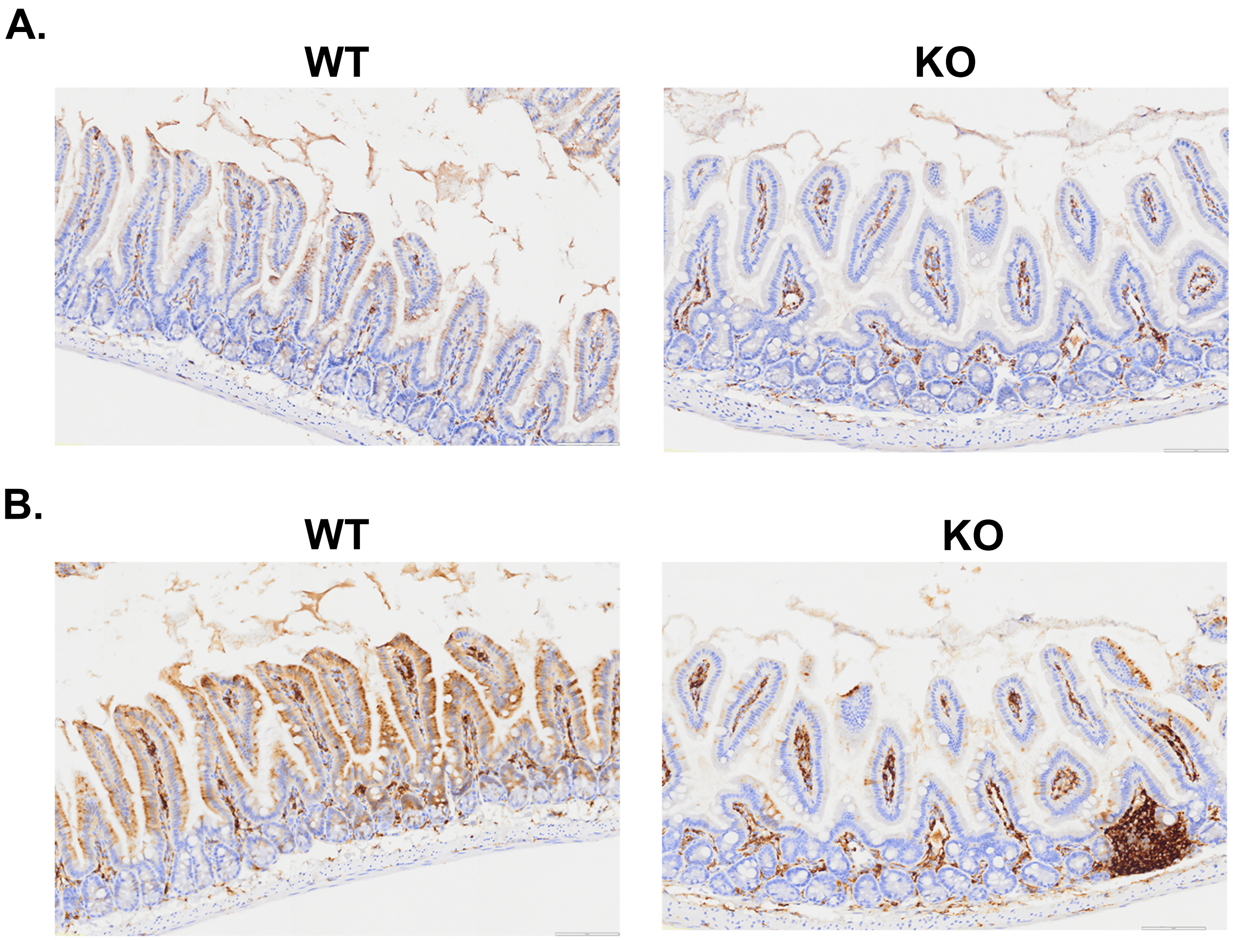

Fig. S5. Immunohistochemistry of H2-Aa (A) and H2-Ab1 (B) in small intestine from WT and KO mice showing expression of these MHCII protein levels in enterocytes. Staining is with hematoxylin (blue) and H2-Aa and H2-Ab1 antibodies (red).

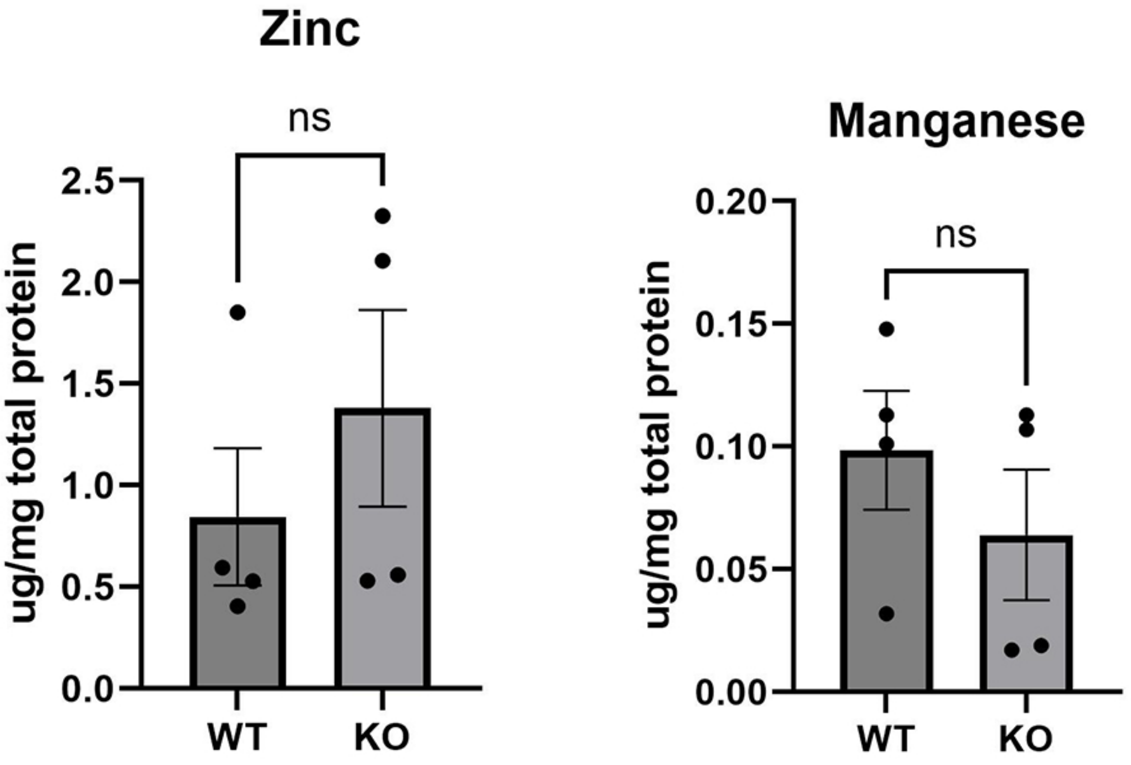

Fig. S6. Zn and Mn concentrations of purified IECs from WT and KO mice as measured by microwave plasma atomic emission spectroscopy.

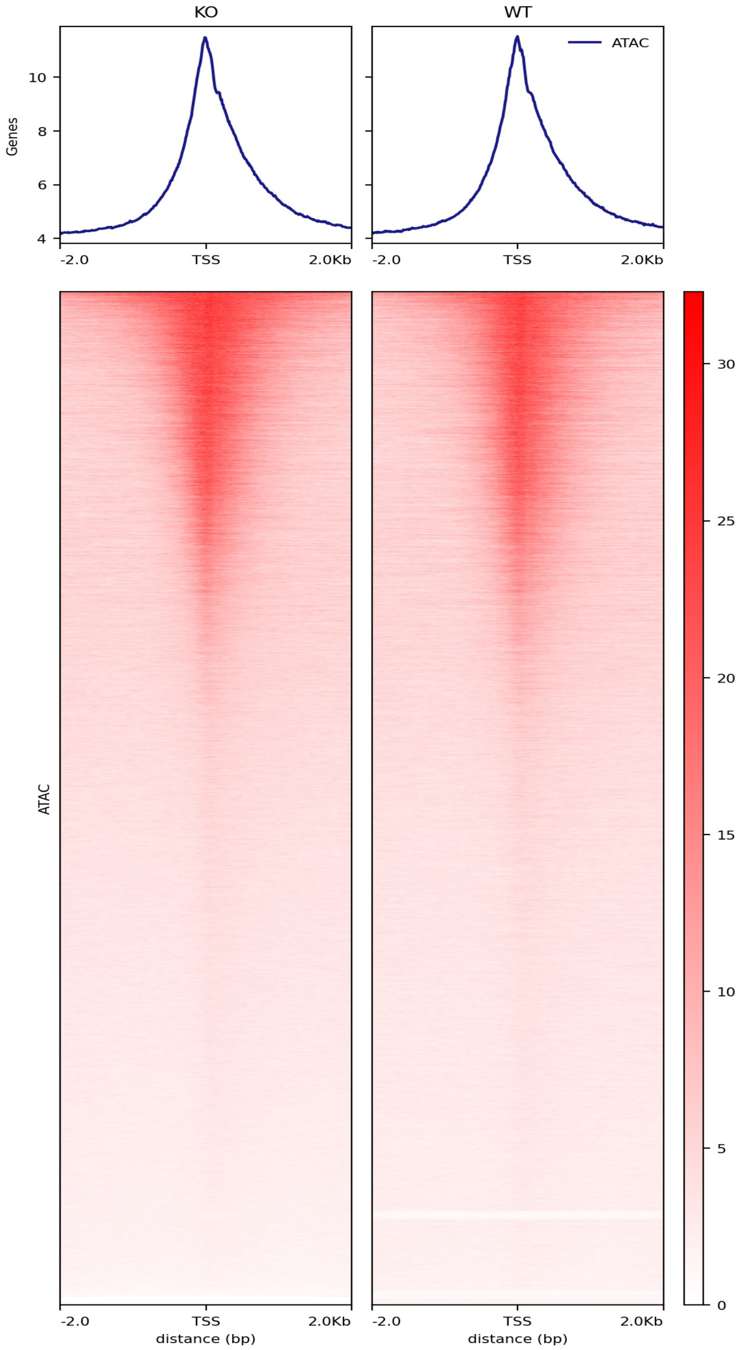

Fig. S7. Heat map showing distribution of closed chromatin from ATAC-sequencing data set.

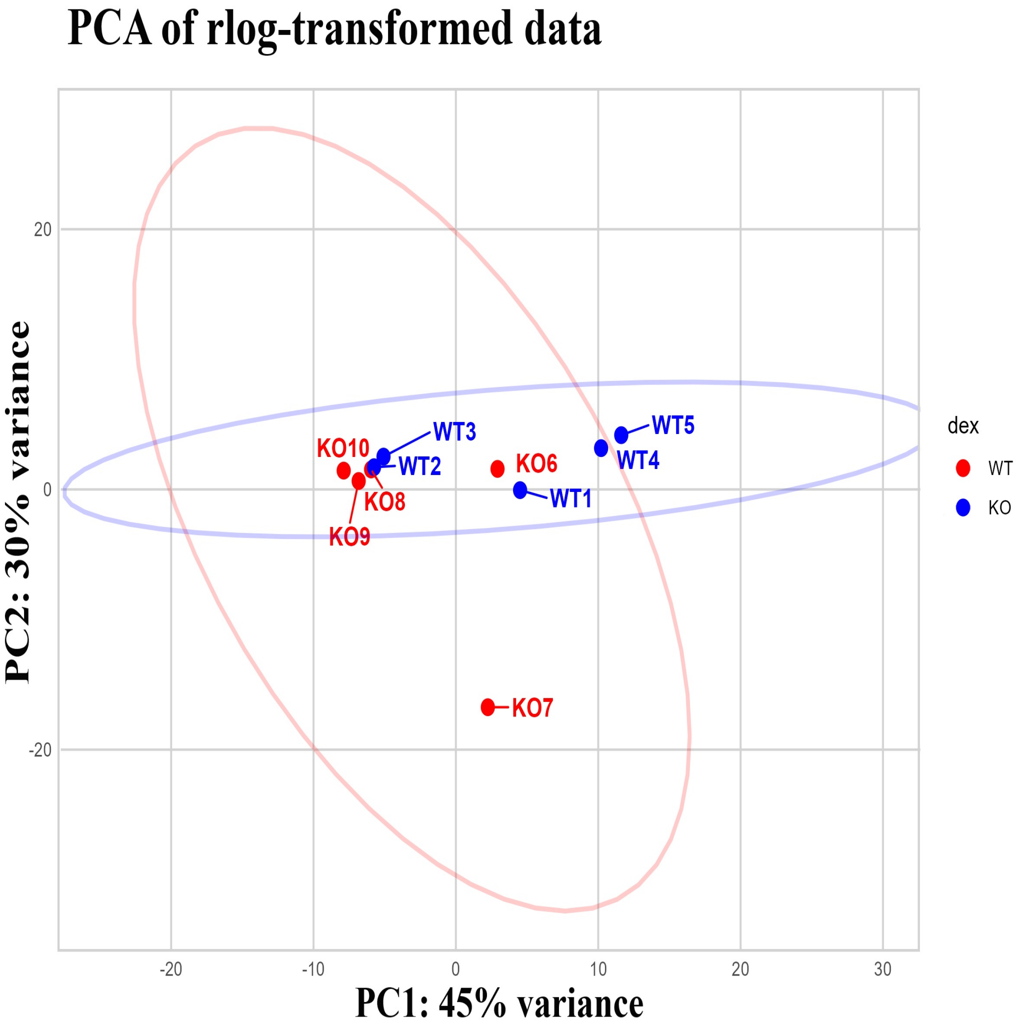

Fig. S8. ATAC-sequencing data set showing PCA and global analysis of all open chromatin from WT and KO mice.

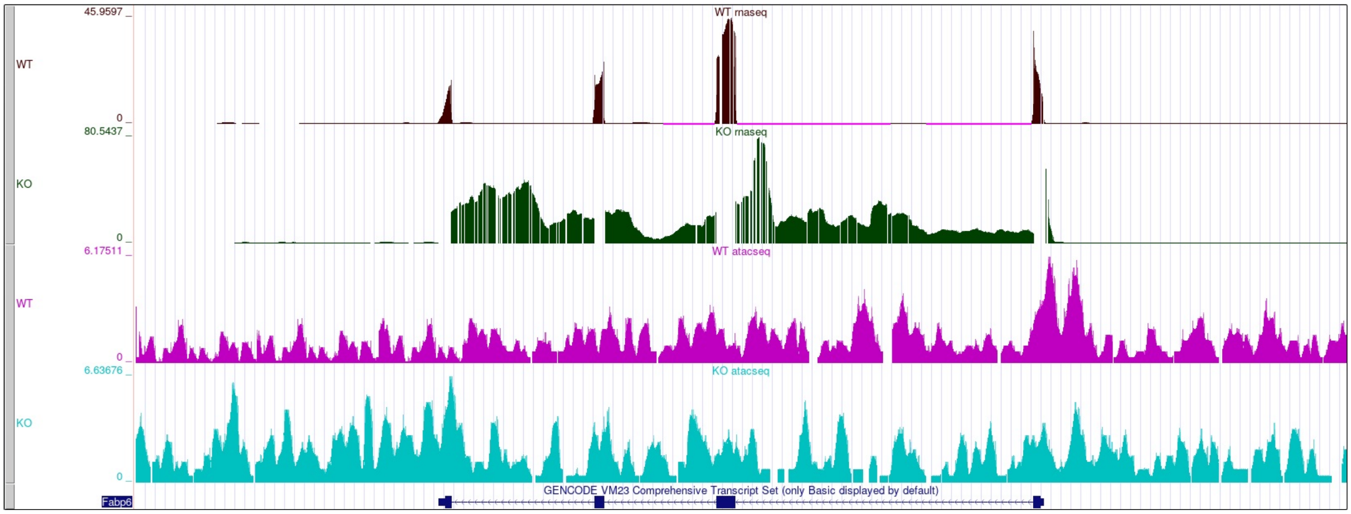

Fig. S9. Peak density of $Fabp6$ promoter. Control example for up-regulated gene.

Table S1. Complete list of microbial species from 16S-rRNA-sequencing from feces of WT and KO mice that have significant changes expressed as KO/WT values (P < 0.05).

	baseMean	log2FoldChange	lfcSE	stat	pvalue	padj
Bacteria_Firmicutes_Bacilli_Lactobacillales_Lactobacillaceae_Lactobacillus_johnsonii	12258.88817	-16.8901	1.358515	-12.4328	1.74E-35	2.43E-34
Bacteria_Bacteroidota_Bacteroidia_Bacteroidales_Bacteroidaceae_Bacteroides_acidifaciens	2710.371107	14.98104	1.338559	11.19192	4.47E-29	3.13E-28
Bacteria_Bacteroidota_Bacteroidia_Bacteroidales_Bacteroidaceae_Bacteroides_caecimuris	534.3657531	12.63846	1.28607	9.827199	8.60E-23	4.01E-22
Bacteria_Firmicutes_Bacilli_Lactobacillales_Lactobacillaceae_Lactobacillus_intestinalis	3088.595044	-14.9013	1.61948	-9.20128	3.54E-20	1.24E-19
Bacteria_Actinobacteriota_Actinobacteria_Bifidobacteriales_Bifidobacteriaceae_Bifidobacterium_pseudolongum	2083.154935	-14.3331	1.669364	-8.58595	9.01E-18	2.52E-17
Bacteria_Bacteroidota_Bacteroidia_Bacteroidales_Bacteroidaceae_Bacteroides_thetaiotaomicron	84.66150678	-9.71085	1.547634	-6.27464	3.50E-10	8.18E-10
Bacteria_Firmicutes_Bacilli_Lactobacillales_Lactobacillaceae_Lactobacillus_gasseri	2124.658248	14.6297	2.573323	5.685138	1.31E-08	2.61E-08
Bacteria_Firmicutes_Bacilli_Erysipelotrichales_Erysipelotrichaceae_Dubosiella_newyorkensis	135.6989421	-10.3928	2.015951	-5.15529	2.53E-07	4.43E-07
Bacteria_Verrucomicrobiota_Verrucomicrobiae_Verrucomicrobiales_Akkermansiaceae_Akkermansia_muciniphila	7090.327894	8.965318	2.099093	4.271044	1.95E-05	3.03E-05
Bacteria_Firmicutes_Clostridia_Peptostreptococcales-Tissierellales_Peptostreptococcaceae_Romboutsia_ilealis	116.14864	-10.1677	2.551071	-3.98567	6.73E-05	9.42E-05
Bacteria_Firmicutes_Bacilli_Erysipelotrichales_Erysipelotrichaceae_Faecalibaculum_rodentium	3778.286141	-2.10986	2.269672	-0.92959	0.352585	0.448744
Bacteria_Firmicutes_Clostridia_Lachnospirales_Lachnospiraceae_Lachnospiraceae_NK4A136_group_bacterium	52.46345367	-0.98818	3.219205	-0.30696	0.758871	0.885349
Bacteria_Actinobacteriota_Actinobacteria_Bifidobacteriales_Bifidobacteriaceae_Bifidobacterium_choerinum	1345.150226	-0.10294	1.922765	-0.05354	0.957303	0.999998
Bacteria_Firmicutes_Bacilli_Lactobacillales_Lactobacillaceae_Lactobacillus_reuteri	4171.895247	-6.70E-07	0.333784	-2.01E-06	0.999998	0.999998
Bacteria_Firmicutes_Bacilli_Lactobacillales_Lactobacillaceae_Lactobacillus_murinus	1357.573492	0.178311	1.733514	0.102861	NA	NA
Bacteria_Firmicutes_Clostridia_Lachnospirales_Lachnospiraceae_Blautia_hansenii	191.3634122	11.15755	1.871205	5.962765	NA	NA

Table S2. Complete list of differentially expressed transcripts from RNA-sequencing of purified intestinal epithelial cells from WT and KO mice. Fold change ± 2.0 (P < 0.05).

	symbol	baseMean	log2FoldChange	lfcSE	stat	pvalue	padj
ENSMUSG00000044976	Wdr72	352.626	11.802	1.347	8.761	1.94E-18	1.98E-16
ENSMUSG00000009588	St6galnac1	215.773	9.243	0.742	12.461	1.22E-35	3.03E-33
ENSMUSG00000095427	Rps2-ps6	104.731	9.086	0.850	10.690	1.14E-26	2.01E-24
ENSMUSG00000061833	Gm6311	58.899	8.736	0.854	10.226	1.51E-24	2.22E-22
ENSMUSG00000075391	Glo1-ps	115.760	7.761	0.610	12.721	4.51E-37	1.21E-34
ENSMUSG00000072476	Rnf26rt	21.098	7.738	0.908	8.521	1.58E-17	1.49E-15
ENSMUSG00000100801	Gm15459	1374.092	7.237	0.525	13.794	2.78E-43	9.91E-41
ENSMUSG00000020405	Fabp6	3305.264	7.156	1.405	5.093	3.52E-07	8.45E-06
ENSMUSG00000090191	9230105E05Rik	11.417	6.857	0.978	7.009	2.39E-12	1.31E-10
ENSMUSG00000091373	Gm8810	8.390	6.408	0.904	7.088	1.36E-12	7.74E-11
ENSMUSG00000091957	Rps2-ps10	596.149	5.916	0.267	22.159	8.49E-109	1.48E-105
ENSMUSG00000052469	Tcp10c	10.726	5.778	0.889	6.496	8.25E-11	3.81E-09
ENSMUSG00000096039	D830030K20Rik	9.437	5.592	0.947	5.906	3.51E-09	1.21E-07
ENSMUSG00000095134	Mid1-ps1	4.608	5.547	1.292	4.295	1.75E-05	0.000257924
ENSMUSG00000089988	Gm16238	4.194	5.411	1.000	5.410	6.30E-08	1.74E-06
ENSMUSG00000037443	Cep85	1220.559	5.367	0.307	17.488	1.76E-68	1.03E-65
ENSMUSG00000073491	Ifi213	13.366	5.217	0.770	6.778	1.22E-11	6.17E-10
ENSMUSG00000032023	Jhy	3.257	5.040	1.015	4.964	6.92E-07	1.54E-05
ENSMUSG00000003053	Cyp2c29	179.135	5.004	0.396	12.628	1.49E-36	3.88E-34
ENSMUSG00000064220	H2ac18	83.639	4.935	1.561	3.161	0.001572456	0.010905987
ENSMUSG00000074194	Zfp791	82.448	4.843	0.326	14.854	6.56E-50	2.79E-47
ENSMUSG00000094326	Gm10045	3.564	4.650	1.073	4.335	1.46E-05	0.000221658
ENSMUSG00000078249	Hmga1b	119.567	4.598	0.314	14.648	1.39E-48	5.51E-46
ENSMUSG00000082051	Gm16072	3.240	4.505	1.247	3.612	0.000303357	0.00289858
ENSMUSG00000095042	Gm12537	2.244	4.505	1.234	3.651	0.000261524	0.002556229
ENSMUSG00000099342	Gm18180	4.401	4.448	1.087	4.091	4.30E-05	0.00055691
ENSMUSG00000049436	Upk1b	45.597	4.356	0.447	9.756	1.74E-22	2.33E-20
ENSMUSG00000083087	Gm11249	10.766	4.255	0.708	6.012	1.83E-09	6.74E-08
ENSMUSG00000066000	Zfp979	1.749	4.150	1.180	3.516	0.000438491	0.003887332
ENSMUSG00000079298	Klrb1b	9.656	4.098	0.712	5.752	8.79E-09	2.90E-07
ENSMUSG00000072844	G530011O06Rik	29.316	4.055	0.562	7.213	5.47E-13	3.22E-11
ENSMUSG00000037849	Ifi206	12.348	4.024	0.593	6.785	1.16E-11	5.86E-10
ENSMUSG00000033491	Prss35	5.795	3.912	0.863	4.531	5.86E-06	0.000101028
ENSMUSG00000016252	Atp5f1e	1239.189	3.875	0.211	18.377	2.02E-75	1.47E-72
ENSMUSG00000027338	Prnd	4.205	3.873	0.948	4.084	4.43E-05	0.00057198
ENSMUSG00000028186	Uox	2.867	3.795	1.084	3.499	0.000466581	0.004082761
ENSMUSG00000037263	Aldh3b3	1.981	3.790	1.150	3.296	0.000980442	0.007470785
ENSMUSG00000036853	Mcoln3	96.238	3.788	0.290	13.056	5.87E-39	1.74E-36
ENSMUSG00000062438	Adam1b	2.832	3.772	1.036	3.642	0.000270789	0.002622791
ENSMUSG00000052819	Best2	2.773	3.751	1.152	3.255	0.001132088	0.008391874
ENSMUSG00000096010	H4c16	7.701	3.741	0.756	4.947	7.55E-07	1.67E-05
ENSMUSG00000028457	Atp8b5	4.868	3.645	0.869	4.195	2.73E-05	0.000377783
ENSMUSG00000030222	Rerg	15.860	3.629	0.576	6.295	3.08E-10	1.30E-08
ENSMUSG00000032878	Ccdc85a	1.754	3.606	1.246	2.894	0.003805877	0.021912661
ENSMUSG00000095753	Igkv4-53	68.077	3.551	0.267	13.291	2.60E-40	8.26E-38
ENSMUSG00000030159	Clec1b	13.483	3.537	0.581	6.089	1.13E-09	4.31E-08
ENSMUSG00000022799	Arhgap31	257.255	3.526	0.179	19.688	2.73E-86	2.80E-83
ENSMUSG00000027832	Ptx3	2.337	3.485	1.021	3.415	0.000637753	0.005241648
ENSMUSG00000029452	Tmem116	154.540	3.484	0.169	20.621	1.79E-94	2.23E-91
ENSMUSG00000076467	Trbv13-1	3.806	3.407	1.308	2.605	0.009186704	0.043530263
ENSMUSG00000040127	Sdr9c7	3.108	3.393	1.082	3.137	0.001709592	0.011682587
ENSMUSG00000021509	Slc25a48	75.922	3.377	1.134	2.978	0.002898253	0.017777443
ENSMUSG00000071230	Npw	2.050	3.305	1.106	2.990	0.002793352	0.017304313
ENSMUSG00000049122	Frmd3	21.614	3.291	0.530	6.204	5.52E-10	2.22E-08
ENSMUSG00000073406	H2-Bl	11434.957	3.197	0.123	25.916	4.37E-148	1.09E-144
ENSMUSG00000029307	Dmp1	47.227	3.135	0.764	4.104	4.07E-05	0.000531575
ENSMUSG00000074361	C5ar2	2.592	3.131	1.042	3.006	0.002645821	0.016537092
ENSMUSG00000085183	Wincr1	2.576	3.126	1.105	2.828	0.004681883	0.025848082

ENSMUSG00000058952	Cfi	26.304	3.113	0.440	7.073	1.51E-12	8.58E-11
ENSMUSG00000017737	Mmp9	80.203	3.074	0.308	9.972	2.01E-23	2.77E-21
ENSMUSG00000026532	Spta1	2.598	3.048	1.126	2.707	0.006790548	0.03441004
ENSMUSG00000092073	Pramel58	5.702	3.039	0.775	3.922	8.78E-05	0.001015554
ENSMUSG00000097789	Ctxnd1	2.391	3.027	1.088	2.782	0.005407364	0.0288979
ENSMUSG00000033377	Palmd	4.780	3.022	0.821	3.683	0.000230638	0.002312606
ENSMUSG00000047414	Flrt2	3.841	3.010	0.866	3.478	0.00050547	0.00436615
ENSMUSG00000096078	Ighv1-62-2	38.771	2.979	0.780	3.818	0.000134708	0.001465166
ENSMUSG00000098387	Pet117	7.725	2.949	1.052	2.802	0.005080037	0.027506887
ENSMUSG00000064057	Scgb3a1	3.170	2.948	1.044	2.823	0.004757547	0.026199527
ENSMUSG00000096326	Ighv1-78	3.853	2.930	1.110	2.639	0.008305121	0.040261732
ENSMUSG00000014813	Stc1	22.891	2.913	0.634	4.596	4.32E-06	7.65E-05
ENSMUSG00000091844	Ccdc168	3.048	2.908	1.012	2.875	0.004045416	0.023026028
ENSMUSG00000025002	Cyp2c55	1838.383	2.894	0.259	11.186	4.80E-29	9.31E-27
ENSMUSG00000078964	Ces1b	24.250	2.862	0.384	7.463	8.47E-14	5.73E-12
ENSMUSG00000042379	Esm1	31.744	2.846	0.539	5.279	1.30E-07	3.38E-06
ENSMUSG00000026069	Il1rl1	142.127	2.845	0.527	5.401	6.65E-08	1.83E-06
ENSMUSG00000096805	Ighv9-1	3.526	2.839	0.908	3.126	0.001774012	0.012022101
ENSMUSG00000041324	Inhba	12.876	2.835	0.666	4.258	2.06E-05	0.000296424
ENSMUSG00000032087	Dscaml1	14.314	2.835	0.438	6.466	1.01E-10	4.60E-09
ENSMUSG00000036356	Csgalnact1	5.649	2.829	0.741	3.819	0.000133823	0.001457357
ENSMUSG00000066721	Zfp575	2.231	2.826	1.033	2.735	0.006234908	0.032173454
ENSMUSG00000019232	Etnppl	5.543	2.783	0.704	3.952	7.74E-05	0.000912751
ENSMUSG00000047592	Nxpe5	5.583	2.782	0.674	4.128	3.66E-05	0.000486772
ENSMUSG00000068794	Col28a1	4.047	2.740	0.767	3.574	0.000351469	0.003258417
ENSMUSG00000062713	Sim2	2.756	2.724	0.932	2.922	0.003480726	0.020493434
ENSMUSG00000021806	Nid2	114.897	2.721	0.180	15.136	9.37E-52	4.09E-49
ENSMUSG00000026065	Slc9a4	4.005	2.720	0.758	3.587	0.000334035	0.00313171
ENSMUSG00000020912	Krt12	527.419	2.691	0.284	9.469	2.82E-21	3.51E-19
ENSMUSG00000020990	Cdkl1	5.324	2.667	0.810	3.294	0.000988165	0.007513237
ENSMUSG00000062006	Rpl34	1392.911	2.664	0.145	18.382	1.83E-75	1.39E-72
ENSMUSG00000024008	Cpne5	26.982	2.659	0.312	8.533	1.42E-17	1.36E-15
ENSMUSG00000048960	Prex2	32.129	2.561	0.488	5.247	1.55E-07	3.98E-06
ENSMUSG00000076665	Ighv7-1	7.022	2.556	0.642	3.982	6.83E-05	0.000819927
ENSMUSG00000078495	Zfp984	80.378	2.553	0.454	5.621	1.90E-08	5.88E-07
ENSMUSG00000028583	Pdpn	15.390	2.548	0.450	5.660	1.51E-08	4.79E-07
ENSMUSG00000084910	C630043F03Rik	17.770	2.547	0.435	5.850	4.92E-09	1.66E-07
ENSMUSG00000050071	Bex1	5.113	2.533	0.828	3.058	0.002227369	0.014411852
ENSMUSG00000063239	Grm4	30.650	2.532	0.405	6.253	4.01E-10	1.64E-08
ENSMUSG00000030325	Klrb1c	6.643	2.527	0.607	4.160	3.18E-05	0.000430767
ENSMUSG00000027208	Fgf7	39.692	2.503	0.398	6.284	3.30E-10	1.39E-08
ENSMUSG00000032500	Dclk3	83.481	2.498	0.249	10.038	1.04E-23	1.46E-21
ENSMUSG00000030154	Klrb1f	2.331	2.492	0.959	2.598	0.009387586	0.044160357
ENSMUSG00000054942	Miga1	260.002	2.491	0.214	11.663	1.98E-31	4.26E-29
ENSMUSG00000049001	Ndnf	6.456	2.489	0.689	3.610	0.000306005	0.002915902
ENSMUSG00000091754	Gm3636	8.588	2.478	0.683	3.627	0.000287223	0.002764083
ENSMUSG00000005681	Apoa2	15.441	2.477	0.680	3.642	0.000270091	0.002617976
ENSMUSG00000029304	Spp1	48.142	2.473	0.694	3.562	0.000367607	0.003380042
ENSMUSG00000021765	Fst	16.871	2.428	0.622	3.901	9.59E-05	0.001091132
ENSMUSG00000022947	Cbr3	21.864	2.391	0.497	4.812	1.50E-06	3.08E-05
ENSMUSG00000043068	Fam89a	5.934	2.389	0.646	3.696	0.000219351	0.002226284
ENSMUSG00000079534	Pwwp4c	4.686	2.379	0.919	2.589	0.009626642	0.044993919
ENSMUSG00000038143	Stox2	88.718	2.369	0.232	10.229	1.47E-24	2.18E-22
ENSMUSG00000082762	Gm12366	9.027	2.350	0.602	3.904	9.47E-05	0.001079868
ENSMUSG00000031849	Comp	17.537	2.334	0.423	5.524	3.31E-08	9.72E-07
ENSMUSG00000023984	Gm20517	7.809	2.325	0.644	3.613	0.000302766	0.002896677
ENSMUSG00000045629	Sh3tc2	3.622	2.324	0.823	2.824	0.00474652	0.026147052
ENSMUSG00000050994	Adgb	4.241	2.316	0.825	2.808	0.004977779	0.027087621
ENSMUSG00000024529	Lox	5.140	2.311	0.633	3.651	0.000261429	0.002556229
ENSMUSG00000070423	Or51e1	4.243	2.307	0.757	3.049	0.002294975	0.014761747
ENSMUSG00000028782	Adgrb2	4.701	2.298	0.762	3.015	0.002570627	0.016172203

ENSMUSG00000049349	Gm5105	9.213	2.294	0.531	4.319	1.57E-05	0.000236122
ENSMUSG00000023073	Slc10a2	125.644	2.282	0.748	3.051	0.002281529	0.014696923
ENSMUSG00000031073	Fgf15	305.264	2.281	0.725	3.146	0.001657856	0.011376258
ENSMUSG00000084890	A830036E02Rik	299.459	2.274	0.271	8.376	5.49E-17	4.99E-15
ENSMUSG00000022122	Ednrb	71.320	2.273	0.402	5.650	1.60E-08	5.05E-07
ENSMUSG00000041633	Kctd12b	23.339	2.259	0.490	4.605	4.12E-06	7.39E-05
ENSMUSG00000094777	H2ac24	138.282	2.242	0.611	3.671	0.00024182	0.002399913
ENSMUSG00000062991	Nrg1	12.108	2.241	0.565	3.968	7.26E-05	0.000861033
ENSMUSG00000081487	Gm13689	13.304	2.239	0.680	3.294	0.000987314	0.007510038
ENSMUSG00000024427	Spry4	26.892	2.204	0.494	4.462	8.12E-06	0.000133734
ENSMUSG00000039438	Ttc36	24.462	2.194	0.508	4.315	1.60E-05	0.000239705
ENSMUSG00000021994	Wnt5a	56.245	2.193	0.391	5.605	2.09E-08	6.40E-07
ENSMUSG00000034488	Edil3	5.762	2.192	0.736	2.979	0.002894822	0.017768953
ENSMUSG00000037010	Apln	8.103	2.190	0.518	4.231	2.33E-05	0.000330473
ENSMUSG00000031841	Cdh13	12.882	2.176	0.515	4.226	2.38E-05	0.000336414
ENSMUSG00000094930	Igkv6-25	75.185	2.165	0.437	4.948	7.51E-07	1.67E-05
ENSMUSG00000057454	Lypd3	5.274	2.159	0.830	2.602	0.00926149	0.043741419
ENSMUSG00000035566	Pcdh17	6.476	2.148	0.736	2.919	0.003512436	0.020635671
ENSMUSG00000000320	Alox12	5.684	2.144	0.755	2.840	0.004516361	0.025140978
ENSMUSG00000046262	Pramel29	7.229	2.134	0.767	2.782	0.005395288	0.028873557
ENSMUSG00000076583	Igkv8-24	91.064	2.133	0.718	2.970	0.002974718	0.01815722
ENSMUSG00000027800	Tm4sf1	57.896	2.132	0.411	5.191	2.10E-07	5.28E-06
ENSMUSG00000030361	Klrb1a	4.741	2.129	0.652	3.265	0.001095014	0.008151667
ENSMUSG00000092243	H2-T25	435.779	2.104	0.173	12.195	3.30E-34	7.67E-32
ENSMUSG00000027870	Hao2	7.080	2.096	0.543	3.861	0.00011282	0.001255212
ENSMUSG00000021732	Fgf10	5.283	2.081	0.680	3.060	0.002215468	0.014350804
ENSMUSG00000073565	Prr16	3.160	2.080	0.809	2.570	0.010180887	0.046993057
ENSMUSG00000004891	Nes	47.018	2.064	0.404	5.114	3.15E-07	7.68E-06
ENSMUSG00000046768	Rhoj	28.457	2.064	0.394	5.233	1.67E-07	4.26E-06
ENSMUSG00000025934	Gsta3	95.356	2.051	0.275	7.460	8.66E-14	5.83E-12
ENSMUSG00000053054	Adh6a	5791.877	2.046	0.183	11.152	7.00E-29	1.33E-26
ENSMUSG00000033082	Clec1a	4.116	2.034	0.699	2.911	0.003599657	0.021030524
ENSMUSG00000037872	Ackr1	4.902	2.031	0.624	3.254	0.001138346	0.008427527
ENSMUSG00000049928	Glp2r	62.378	2.001	0.326	6.141	8.20E-10	3.19E-08
ENSMUSG00000072612	Gm10382	15.459	-2.003	0.379	-5.279	1.30E-07	3.38E-06
ENSMUSG00000039518	Cdsn	7.381	-2.004	0.548	-3.653	0.000259406	0.002541216
ENSMUSG00000036928	Stag3	3.048	-2.004	0.771	-2.600	0.009327809	0.043962086
ENSMUSG00000057897	Camk2b	299.666	-2.005	0.147	-13.685	1.26E-42	4.30E-40
ENSMUSG00000022504	Ciita	786.148	-2.006	0.270	-7.429	1.09E-13	7.27E-12
ENSMUSG00000030762	Aqp8	33.785	-2.014	0.402	-5.010	5.44E-07	1.24E-05
ENSMUSG00000028738	Tas1r2	6.637	-2.016	0.591	-3.409	0.000651963	0.00532584
ENSMUSG00000092517	Art2a	57.130	-2.033	0.274	-7.421	1.16E-13	7.70E-12
ENSMUSG00000010492	Uckl1os	40.518	-2.042	0.292	-6.985	2.84E-12	1.54E-10
ENSMUSG00000032221	Mns1	12.120	-2.042	0.442	-4.617	3.90E-06	7.06E-05
ENSMUSG00000020401	Garin3	3.991	-2.061	0.738	-2.794	0.005209866	0.028100309
ENSMUSG00000021730	Hcn1	5.652	-2.077	0.579	-3.585	0.000337454	0.003148548
ENSMUSG00000023484	Prph	8.606	-2.078	0.619	-3.358	0.000785704	0.006226075
ENSMUSG00000021091	Serpina3n	24.382	-2.079	0.666	-3.125	0.00178075	0.012053724
ENSMUSG00000028037	Ifi44	76.654	-2.084	0.636	-3.279	0.0010402	0.007810223
ENSMUSG00000001473	Tubb6	152.774	-2.086	0.179	-11.623	3.17E-31	6.74E-29
ENSMUSG00000059654	Reg1	3629.654	-2.096	0.607	-3.452	0.000556381	0.004701709
ENSMUSG00000052031	Tagap1	168.347	-2.113	0.129	-16.418	1.42E-60	7.30E-58
ENSMUSG00000031860	Pbx4	36.990	-2.114	0.296	-7.137	9.56E-13	5.52E-11
ENSMUSG00000030630	Fah	43.273	-2.125	0.304	-7.002	2.52E-12	1.38E-10
ENSMUSG00000097099	Gm9917	428.984	-2.131	0.134	-15.876	9.23E-57	4.60E-54
ENSMUSG00000037797	Adh4	385.131	-2.154	0.303	-7.104	1.21E-12	6.94E-11
ENSMUSG00000037860	Aim2	260.396	-2.166	0.143	-15.148	7.84E-52	3.51E-49
ENSMUSG00000049532	Sall2	24.608	-2.173	0.379	-5.737	9.64E-09	3.16E-07
ENSMUSG00000076594	Igkv6-13	11.963	-2.177	0.587	-3.706	0.000210752	0.002154035
ENSMUSG00000069873	4930438A08Rik	8.560	-2.179	0.635	-3.433	0.00059772	0.004964032
ENSMUSG00000076666	Ighv14-4	6.030	-2.191	0.803	-2.729	0.00635938	0.032670895

ENSMUSG00000066687	Zbtb16	13.918	-2.206	0.370	-5.956	2.59E-09	9.16E-08
ENSMUSG00000022039	Adam2	6.048	-2.225	0.626	-3.556	0.000376806	0.003438406
ENSMUSG00000025004	Cyp2c40	71.160	-2.226	0.807	-2.757	0.005827822	0.030423533
ENSMUSG00000031803	B3gnt3	4137.681	-2.233	0.157	-14.224	6.55E-46	2.48E-43
ENSMUSG00000060586	H2-Eb1	12724.585	-2.250	0.245	-9.168	4.84E-20	5.59E-18
ENSMUSG00000096515	Igkv14-100	8.477	-2.263	0.574	-3.943	8.04E-05	0.000941793
ENSMUSG00000086228	Ubap1l	4.507	-2.264	0.647	-3.500	0.000465361	0.004074124
ENSMUSG00000076526	Igkv12-98	4.916	-2.275	0.655	-3.471	0.000517998	0.004445461
ENSMUSG00000049555	Tmie	23.407	-2.276	0.376	-6.051	1.44E-09	5.35E-08
ENSMUSG00000021702	Thbs4	4.930	-2.277	0.772	-2.950	0.003177231	0.019060113
ENSMUSG00000020926	Adam11	16.464	-2.279	0.391	-5.822	5.80E-09	1.94E-07
ENSMUSG00000060613	Cyp2c70	4.082	-2.307	0.704	-3.274	0.001059918	0.007924194
ENSMUSG00000029605	Oas1b	112.699	-2.329	0.230	-10.107	5.12E-24	7.32E-22
ENSMUSG00000021609	Slc6a3	55.478	-2.332	0.468	-4.989	6.08E-07	1.37E-05
ENSMUSG00000074968	Ano3	60.151	-2.354	0.280	-8.408	4.18E-17	3.84E-15
ENSMUSG00000043664	Tmem221	3.777	-2.354	0.737	-3.195	0.001398979	0.009939758
ENSMUSG00000049892	Rasd1	38.519	-2.369	0.314	-7.533	4.95E-14	3.48E-12
ENSMUSG00000002289	Angptl4	1331.996	-2.375	0.192	-12.384	3.18E-35	7.81E-33
ENSMUSG00000079547	H2-DMb1	2408.290	-2.380	0.292	-8.163	3.26E-16	2.79E-14
ENSMUSG00000024610	Cd74	50324.639	-2.392	0.388	-6.158	7.36E-10	2.89E-08
ENSMUSG00000038583	Pln	4.396	-2.406	0.858	-2.805	0.005030595	0.027298447
ENSMUSG00000070532	Ccdc190	18.498	-2.418	0.338	-7.155	8.38E-13	4.88E-11
ENSMUSG00000069456	Rdh16	1046.950	-2.422	0.158	-15.310	6.53E-53	3.08E-50
ENSMUSG00000073785	Krtap5-5	2.472	-2.422	0.937	-2.586	0.009712732	0.045275077
ENSMUSG00000032690	Oas2	213.796	-2.439	0.234	-10.430	1.80E-25	2.89E-23
ENSMUSG00000047507	Baiap3	135.080	-2.446	0.232	-10.551	5.00E-26	8.24E-24
ENSMUSG00000024222	Fkbp5	605.408	-2.466	0.183	-13.486	1.88E-41	6.19E-39
ENSMUSG00000086589	Gm12915	5.014	-2.468	0.714	-3.458	0.000544514	0.004630091
ENSMUSG00000073421	H2-Ab1	26866.231	-2.493	0.378	-6.595	4.26E-11	2.05E-09
ENSMUSG00000004360	9330159F19Rik	2.533	-2.501	0.970	-2.578	0.009948323	0.046225146
ENSMUSG00000083332	Gm7599	3.067	-2.515	0.797	-3.154	0.001609382	0.011100351
ENSMUSG00000028979	Masp2	3.571	-2.532	0.769	-3.292	0.000995954	0.007552726
ENSMUSG00000096904	Lamtor3l	7.329	-2.537	0.750	-3.381	0.00072331	0.005800101
ENSMUSG00000096629	Gm3383	2.587	-2.544	0.856	-2.973	0.002951496	0.018040709
ENSMUSG00000033576	Apol6	10.109	-2.547	0.522	-4.876	1.08E-06	2.29E-05
ENSMUSG00000048655	Ccdc169	3.209	-2.555	0.903	-2.828	0.004681244	0.025848082
ENSMUSG00000042942	Greb1l	32.204	-2.577	0.310	-8.303	1.01E-16	9.10E-15
ENSMUSG00000095335	Igkv3-5	152.739	-2.578	0.802	-3.216	0.001298787	0.009372849
ENSMUSG00000044229	Nxpe4	6.891	-2.584	0.614	-4.206	2.60E-05	0.000361353
ENSMUSG00000040680	Kremen2	19.370	-2.614	0.414	-6.311	2.76E-10	1.18E-08
ENSMUSG00000068758	Il3ra	90.536	-2.624	0.158	-16.596	7.42E-62	4.05E-59
ENSMUSG00000046082	Tmem174	8.306	-2.629	0.590	-4.455	8.40E-06	0.000137742
ENSMUSG00000076480	Trbv29	3.346	-2.638	0.880	-2.999	0.00270508	0.016841151
ENSMUSG00000002475	Abhd3	7192.207	-2.671	0.131	-20.443	6.99E-93	8.13E-90
ENSMUSG00000036749	Pramel5	7.816	-2.671	0.583	-4.584	4.57E-06	8.06E-05
ENSMUSG00000026904	Slc4a10	157.571	-2.679	0.259	-10.328	5.27E-25	7.86E-23
ENSMUSG00000079339	Ifit1bl1	1211.973	-2.687	0.216	-12.462	1.21E-35	3.03E-33
ENSMUSG00000039155	Cdh26	10.155	-2.688	0.468	-5.745	9.17E-09	3.02E-07
ENSMUSG00000079015	Serpina1c	17.319	-2.701	0.521	-5.182	2.19E-07	5.50E-06
ENSMUSG00000036594	H2-Aa	21488.990	-2.733	0.264	-10.345	4.41E-25	6.63E-23
ENSMUSG00000058728	Cd300c	2.413	-2.746	1.024	-2.682	0.00731769	0.036613619
ENSMUSG00000019577	Pdk4	759.061	-2.757	0.424	-6.504	7.81E-11	3.63E-09
ENSMUSG00000061843	Vmn1r-ps32	4.863	-2.801	0.826	-3.392	0.000692933	0.005610635
ENSMUSG00000022857	Tmprss15	45.190	-2.895	0.899	-3.222	0.001271856	0.009205136
ENSMUSG00000021647	Cartpt	2.670	-2.901	0.986	-2.944	0.003243462	0.019351029
ENSMUSG00000001168	Oas1h	2.641	-2.921	0.951	-3.071	0.002130641	0.013899324
ENSMUSG00000037583	Nr0b2	180.688	-2.937	0.307	-9.583	9.41E-22	1.21E-19
ENSMUSG00000066867	Oas1e	22.215	-2.952	0.402	-7.338	2.17E-13	1.38E-11
ENSMUSG00000058064	Gm10036	16.435	-2.972	0.392	-7.582	3.40E-14	2.43E-12
ENSMUSG00000067058	Rps15a-ps5	2.749	-2.992	1.004	-2.981	0.002873321	0.017668041
ENSMUSG00000058626	Capn11	32.026	-3.009	1.025	-2.935	0.00333584	0.019793937

ENSMUSG00000034681	Rnps1	495.261	-3.015	0.104	-29.021	3.60E-185	1.57E-181
ENSMUSG00000087382	Ctcflos	14.941	-3.040	0.536	-5.671	1.42E-08	4.52E-07
ENSMUSG00000061167	Rpl15-ps3	63.626	-3.123	0.843	-3.705	0.000211641	0.002159328
ENSMUSG00000021850	Ccdc198	4.632	-3.176	0.882	-3.599	0.000319995	0.003024836
ENSMUSG00000039716	Dock3	21.045	-3.183	0.375	-8.495	1.98E-17	1.85E-15
ENSMUSG00000094724	Rnaset2b	1324.990	-3.223	0.136	-23.767	7.25E-125	1.58E-121
ENSMUSG00000066368	Actl11	1.761	-3.257	1.201	-2.711	0.006701548	0.034047997
ENSMUSG00000048758	Rpl29	1090.944	-3.264	0.221	-14.773	2.17E-49	9.03E-47
ENSMUSG00000073437	D330041H03Rik	264.228	-3.315	0.172	-19.272	9.25E-83	8.97E-80
ENSMUSG00000038663	Fsd2	1.798	-3.331	1.126	-2.958	0.003096844	0.018712913
ENSMUSG00000030703	Gdpd3	171.241	-3.405	0.158	-21.576	3.02E-103	4.40E-100
ENSMUSG00000054052	Rdh19	6.323	-3.412	0.679	-5.027	4.97E-07	1.15E-05
ENSMUSG00000035186	Ubd	301.686	-3.424	0.425	-8.060	7.66E-16	6.40E-14
ENSMUSG00000079019	Insl3	30.925	-3.465	0.393	-8.821	1.14E-18	1.18E-16
ENSMUSG00000079492	H2-T27	3583.174	-3.465	0.148	-23.339	1.78E-120	3.46E-117
ENSMUSG00000071322	Tcp10a	36.725	-3.480	0.387	-9.003	2.19E-19	2.42E-17
ENSMUSG00000028571	Cyp2j13	21.259	-3.507	0.479	-7.328	2.33E-13	1.48E-11
ENSMUSG00000043020	Dnai3	12.779	-3.559	0.510	-6.972	3.12E-12	1.69E-10
ENSMUSG00000054672	Scart2	3.971	-3.601	1.047	-3.438	0.000586634	0.004895252
ENSMUSG00000010825	Grid2ip	2.145	-3.616	1.228	-2.945	0.003225755	0.019258553
ENSMUSG00000035184	Fam124a	2.233	-3.648	1.210	-3.015	0.002570743	0.016172203
ENSMUSG00000090215	Trim34b	24.925	-3.649	0.371	-9.826	8.74E-23	1.18E-20
ENSMUSG00000089694	Nat8f7	19.350	-3.670	1.204	-3.048	0.002301797	0.014783835
ENSMUSG00000036242	Armh4	22.594	-3.850	0.500	-7.696	1.41E-14	1.04E-12
ENSMUSG00000079391	Gm2974	5.036	-3.945	0.889	-4.437	9.10E-06	0.000147952
ENSMUSG00000021187	Tc2n	65.299	-3.952	0.322	-12.268	1.35E-34	3.17E-32
ENSMUSG00000038193	Hand2	3.863	-3.957	1.149	-3.444	0.000574177	0.004816628
ENSMUSG00000031698	Mylk3	15.832	-4.010	0.545	-7.353	1.93E-13	1.24E-11
ENSMUSG00000047564	Krtap3-1	2.116	-4.086	1.139	-3.586	0.000335477	0.003138488
ENSMUSG00000040189	Odad1	65.714	-4.158	1.196	-3.477	0.000506736	0.004370594
ENSMUSG00000079364	Gm3558	5.840	-4.195	0.977	-4.293	1.76E-05	0.00025978
ENSMUSG00000046101	Mcmdc2	6.114	-4.229	0.875	-4.835	1.33E-06	2.78E-05
ENSMUSG00000085302	Trav8-2	2.335	-4.266	1.264	-3.375	0.000736992	0.005896274
ENSMUSG00000030218	Mgp	2.392	-4.272	1.375	-3.107	0.001890474	0.012644447
ENSMUSG00000072324	Gm8420	2.034	-4.597	1.228	-3.744	0.000181268	0.001888065
ENSMUSG00000084403	Rps15a-ps8	2.144	-4.654	1.071	-4.344	1.40E-05	0.000214711
ENSMUSG00000020017	Hal	161.227	-5.157	0.233	-22.117	2.15E-108	3.41E-105
ENSMUSG00000047347	Tdg-ps	20.301	-5.198	0.617	-8.427	3.55E-17	3.29E-15
ENSMUSG00000085882	2610507I01Rik	31.968	-5.205	0.490	-10.631	2.14E-26	3.66E-24
ENSMUSG00000066362	Rps13-ps1	99.783	-5.259	0.289	-18.223	3.41E-74	2.38E-71
ENSMUSG00000063953	Amd2	21.732	-5.294	0.588	-8.997	2.32E-19	2.55E-17
ENSMUSG00000103922	Gm6123	68.081	-5.295	0.384	-13.789	2.96E-43	1.04E-40
ENSMUSG00000046952	Gm5815	3.309	-5.300	1.044	-5.079	3.80E-07	9.06E-06
ENSMUSG00000080893	Ndufa12-ps	74.610	-5.304	0.366	-14.476	1.71E-47	6.62E-45
ENSMUSG00000021747	Cfap20dc	3.579	-5.392	1.014	-5.318	1.05E-07	2.78E-06
ENSMUSG00000090691	Gm3667	5.822	-5.591	0.921	-6.067	1.30E-09	4.89E-08
ENSMUSG00000012187	Mogat1	20.517	-5.712	0.762	-7.498	6.49E-14	4.48E-12
ENSMUSG00000076525	Igkv1-99	30.879	-5.777	1.167	-4.950	7.42E-07	1.65E-05
ENSMUSG00000022899	Slc15a2	150.495	-5.833	1.291	-4.520	6.19E-06	0.000105587
ENSMUSG00000032038	St3gal4	28508.597	-5.930	0.209	-28.410	1.54E-177	4.49E-174
ENSMUSG00000026535	Ifi202b	6.617	-6.297	0.928	-6.788	1.13E-11	5.77E-10
ENSMUSG00000072849	Serpina1e	8.105	-6.571	0.935	-7.026	2.12E-12	1.17E-10
ENSMUSG00000081087	Rps15a-ps7	38.091	-6.939	0.743	-9.345	9.23E-21	1.10E-18
ENSMUSG00000066107	Larp7-ps	11.056	-7.021	0.882	-7.963	1.68E-15	1.36E-13
ENSMUSG00000096574	Gm2956	12.102	-7.159	0.879	-8.145	3.79E-16	3.20E-14
ENSMUSG00000038311	2410017I17Rik	81.938	-7.733	0.661	-11.691	1.42E-31	3.11E-29
ENSMUSG00000068417	Pnp2	1295.305	-7.928	0.279	-28.461	3.56E-178	1.24E-174
ENSMUSG00000051262	Nat8f3	66.541	-8.152	1.626	-5.013	5.37E-07	1.23E-05
ENSMUSG00000073402	H2-T26	3314.154	-8.648	0.173	-50.031	0	0
ENSMUSG00000068396	Rpl34-ps1	406.541	-8.665	0.416	-20.834	2.11E-96	2.83E-93
ENSMUSG00000059040	Eno1b	5261.106	-10.631	0.283	-37.537	0	0

References

1. TB Aydemir, et al., Zinc transporter zip14 functions in hepatic zinc, iron and glucose homeostasis during the innate immune response (endotoxemia). *PLoS One* **7**, e48679 (2012).
2. FR Jimenez-Rondan, et al., Enterocyte-specific deletion of metal transporter zip14 (slc39a14) alters intestinal homeostasis through epigenetic mechanisms. *Am J Physiol Gastrointest Liver Physiol* **324**, G159–g176 (2023).
3. E Aughey, L Grant, BL Furman, WF Dryden, The effects of oral zinc supplementation in the mouse. *J. Comp. Pathol.* **87**, 1–14 (1977).
4. EL Collins, et al., Inhibition of SOCS1 Lethal Autoinflammatory Disease Correlated to Enhanced Peripheral Foxp3+ Regulatory T Cell Homeostasis. *The J. Immunol.* **187**, 2666–2676 (2011).
5. Y Ge, M Zadeh, M Mohamadzadeh, Dissociation and flow cytometric isolation of murine intestinal epithelial cells for multi-omic profiling. *STAR Protoc.* **4**, 101936 (2023).
6. J Kim, et al., Deletion of metal transporter zip14 (slc39a14) produces skeletal muscle wasting, endotoxemia, mef2c activation and induction of mir-675 and hspb7. *Sci Rep* **10**, 4050 (2020).
7. H Haase, S Hebel, G Engelhardt, L Rink, Flow cytometric measurement of labile zinc in peripheral blood mononuclear cells. *Anal. Biochem.* **352**, 222–230 (2006).
8. EM Eshleman, et al., Intestinal epithelial hdac3 and mhc class ii coordinate microbiota-specific immunity. *J Clin Invest* **133** (2023).
